# Promiscuous, persistent and problematic: insights into current enterococcal genomics to guide therapeutic strategy

**DOI:** 10.1186/s12866-024-03243-2

**Published:** 2024-03-28

**Authors:** David Hourigan, Ewelina Stefanovic, Colin Hill, R. Paul Ross

**Affiliations:** 1 APC Microbiome Ireland, Biosciences Institute, Biosciences Research Institute, College Rd, University College, Cork, Ireland; 2 School of Microbiology, University College Cork, College Rd, University College, Cork, Ireland; 3 Teagasc Food Research Centre, Moorepark, Moorepark West, Fermoy, Co. Cork, Ireland

**Keywords:** Enterococcus, Antimicrobial resistance (AMR), Bacteriocins, Bacteriophage, Synergy, Colonisation-resistance

## Abstract

Vancomycin-resistant enterococci (VRE) are major opportunistic pathogens and the causative agents of serious diseases, such as urinary tract infections and endocarditis. VRE strains mainly include species of *Enterococcus faecium* and *E. faecalis* which can colonise the gastrointestinal tract (GIT) of patients and, following growth and persistence in the gut, can transfer to blood resulting in systemic dissemination in the body. Advancements in genomics have revealed that hospital-associated VRE strains are characterised by increased numbers of mobile genetic elements, higher numbers of antibiotic resistance genes and often lack active CRISPR-Cas systems. Additionally, comparative genomics have increased our understanding of dissemination routes among patients and healthcare workers. Since the efficiency of currently available antibiotics is rapidly declining, new measures to control infection and dissemination of these persistent pathogens are urgently needed. These approaches include combinatory administration of antibiotics, strengthening colonisation resistance of the gut microbiota to reduce VRE proliferation through commensals or probiotic bacteria, or switching to non-antibiotic bacterial killers, such as bacteriophages or bacteriocins. In this review, we discuss the current knowledge of the genomics of VRE isolates and state-of-the-art therapeutic advances against VRE infections.

## Background

Vancomycin-resistant enterococci often represent multi-drug resistance strains and pose a recurring and lethal risk to patients in healthcare facilities. Hypermutable enterococci of clinical interest, such as *Enterococcus faecium*, often lack CRISPR systems, resulting in the promiscuous acquisition of exogenous DNA and the ability to mutate and persist in challenging niches, such as the healthcare setting. Considered a model of adaptability to its environment due to genomic plasticity, clinical *Enterococcus* spp. have accumulated resistance genes to aminoglycosides, quinupristin/dalfopristin, linezolid, macrolide, phenicols, tetracyclines and, most notoriously, vancomycin. Vancomycin is still used as a last-line treatment to treat *Clostridioides difficile* infection and cases of vancomycin resistant *C. difficile* (VRCD) are on the rise. Recently enterococci have been shown to enhance *C. difficile* pathogenesis substantiating their role in poly-microbial infections. As of June 2021, a novel mechanism of vancomycin resistance was discovered in *E. faecium* (VREfm). As of October 2022, the first case of a plasmid-borne *VanD* resistance operon was identified in *E. faecium* nested within a transposon. The IS256 family transposase has similarity to a transposase of *C. difficile* origin. Since the turn of the century, an increasing incidence of multidrug-resistant (MDR) *E. faecium* has been observed, a trend that continues to be observed across Europe in recent years (2021–2023).

Recent genomic analyses have highlighted the multifaceted role plasmids, mobile genetic elements, and their dissemination play in the advancement of VREfm to become a clinical pathogen. A phylogenomic analysis accounting for recombination events, a process which results in genomic admixture of clones, suggests the most clinically significant clade A1 emerged from clade A2, both descendent of the community clade B, and that new genomic forms arise through genomic exchange between these members resulting in novel lineages. Similarly, the plasmid population among VREfm is a key driver in the distribution of antimicrobial resistance (AMR) genes, and specific plasmid profiles can be used to suggest the originating source of enterococcal isolates.

Current gold standard treatments to treat VREfm require patient-tailored regimens based on the susceptibility pattern of isolates and the degree of infection. Cases of daptomycin resistant Enterococcus (DRE) are on the rise, cases of DRE transmission within the healthcare setting have been identified and DRE have been isolated from daptomycin naïve patients. Clinical isolates are also able to undergo selective genomic rearrangements to confer rapid resistance.

A recent review recommended methods of infection control and diagnostics of VRE, such as intensified cleaning procedures, antibiotic stewardship, and genomic surveillance. The scope of containment needs to be expanded due to the potential for the dissemination of MDR strains in the food chain and community spread. García-Solache and Rice [1] suggest *Enterococcus* has become a model of adaptability to its environment and summarise the resistance profile of *Enterococcus* to a wide array of antibiotics. Ahmed and Baptiste [2] explore the mechanisms of vancomycin resistance among enterococcal isolates and discuss the evidence of the link between VRE and Enterococcal spp. of animal origin.

In this review, we describe the journey of *Enterococcus* spp. to become a nosocomial pathogen from its commensal background, the traits associated with clinical isolates and review current genomic and phylogenomic literature surrounding clonal epidemiology, discuss novel mechanisms of resistance and the dissemination of resistance genes *via* plasmids and mobile genetic elements. From these genomic insights, we discuss strategies of therapeutic intervention based on aetiology, including combinations of antibiotics, bacteriocins, probiotics, bacteriophage (phage) therapy and strengthening colonisation resistance of the gut microbiota against VRE.

### Statement

The aim of this review is to highlight the multifaceted traits relevant to clinical *Enterococcus* spp. and their community counterparts, then discuss the use of antibiotic and non-antibiotic strategies to combat infection and dissemination. We also discuss how genomics can improve these strategies.

The scope of this review is to assess the current genomic literature surrounding resistance, mobile genetic elements, and epidemiology and to discuss microbiological strategies guided by genomics to prevent colonisation by carrier VRE or treat infection.

VRE represent MDR strains routinely capable of genomic exchange, as seen with the discovery of novel resistance mechanisms and newly vectorised resistance operons. Increasing resistance, including treatment naïve resistance, is being documented to current gold-standard antibiotic treatments.

Recent publications in the field suggest *E. faecium* portray increased incidence, acquisition of novel resistance mechanisms and dissemination. This review is timely as it collates the microbiological tools which will be required to treat or prevent enterococcal infection in the clinical setting and reduce community spread. Strategies discussed also include prophylactic colonisation resistance and probiotics.

## Main text

### Introduction

Enterococci are gram-positive, chain-forming, non-spore-forming, facultative anaerobic lactic acid bacteria (LAB), commonly isolated from the gastrointestinal tract (GIT) of humans and animals [3]. They delineated from *Vagococcus* ~ 500 million years ago, have co-evolved with animal territorialisation and have associated heavily with the mammalian GIT [4]. The human gut microbiota hosts approximately < 0.1% enterococci [4]. Previously classified as part of the group D *Streptococcus* based on the Lancefield serologic typing system, they were acknowledged as a separate genus in the 1980s, *Enterococcus* [5]. This genus currently contains 83 species [6]. Comparative genomic analysis of 37 *Enterococcus* strains revealed that this genus represents a group with variation in GC content (34–45%) and genome size (2.31 Mbp to 5.5 Mbp) [4, 7]. Functional analysis of the pan-genome highlights the flux of niche-specific genes (NSG) over time, where the greatest flux of annotatable genes is associated with carbon utilisation, phosphotransferase systems (PTS) and transcriptional regulation [4]. This indicates the evolution of *Enterococcus* coupled with horizontal gene transfer (HGT) events, selective pressure, and niche transition. Enrichment of genes involved in cell wall modification, de novo purine biosynthesis and stress response suggests adaptability to niche diversification and phenotypic resilience due to genomic plasticity resulting in genus diversification [4].

The genus, previously as part of *Streptococcus,* was first described as “hardy” in 1899. An analysis of phenotypic growth in the presence of stressors associated with the hospital environment found ubiquitous resistance to β-lactam antibiotics and common disinfectants. *E. faecium* and *E. faecalis* were some of the most desiccant and starvation resistant, respectively [4, 8]. The enterococcal pangenome contains ~ 29,545 gene families and grows continuously, pointing to an open pangenome suggesting gene exchange within and between species [7]. However, this is not the case for all species within the genus, as *E. faecium* and *E. faecalis* have open and closed pan-genomes, respectively [9, 10]. Habitats drive the evolution of *Enterococcus*, and genetic relationships are more similar in strains that come from the same environment [11]. Phylogenetics of the core-genome show that human and mammalian isolates are dispersed in branches of *E. faecium*, *E. dispar* and *E. pallens*, while plant and bird isolates are mainly in the *E. casseliflavus* branch suggesting dissemination of the genus among mammals [7].

Enterococci are used in the fermentation of certain types of cheeses (e.g. traditional European cheeses) and meat products (e.g. fermented sausage) [12]. They are also causative agents of food spoilage, mainly of cooked meats [13]. Enterococci have also been successfully used as probiotics but remain controversial for such applications given their genetic promiscuity and relatedness to pathogenic strains [13, 14]. They are part of the commensal microbiota in the gut but can cause infectious diseases, such as endocarditis, urinary tract infections (UTI) and bacteraemia. The two species most associated with invasive infection are *E. faecium* and *E. faecalis*. Treatment of the resulting diseases is often complex due to their resistance to commonly used chemotherapeutic agents [5]. The first documented use of the term “enterococcus” in 1899 highlighted the bacterium’s ability to become pathogenic; presently, *E. faecium* represents a pathobiont currently a threat to global health [15]. One of the “hottest” issues regarding pathogenic enterococci is the emergence of multidrug resistant (MDR) strains, leading to enterococci becoming the 2^nd^ most causative agent of hospital-acquired infections (HAI) [1]. Enterococci also enhance the pathogenesis of *Clostridioides difficile* suggesting their role in poly microbial infections [16].

On average, *E. faecium* clinical isolates (CL) harbour 10 resistance genes, including vancomycin, aminoglycoside, macrolide-lincosamide-streptogramin, and tetracycline [17]. Daptomycin, a first-line treatment for VRE, was recently shown to select for off-target resistance within the human after intravenous treatment [18]. Non-synonymous mutations conferring resistance to daptomycin are detected globally, indicating the emergence of resistant mutants due to local selective pressures. However, these do not correlate significantly with vancomycin resistance genes [17]. Evidence is also emerging of small colony variants (SCV) among *E. faecium* and *E. faecalis* species, a phenomenon usually associated with increased robustness, antibiotic resistance and recurrent infections. To date, described cases of SCVs among enterococci are vancomycin susceptible [19, 20].

This review provides detail on the mechanisms of vancomycin resistance in enterococci. We examine the evolutionary relationships between hospital-associated pathogenic enterococci and their community counterparts based on genomics and present the likely routes of transmission based on this data. Finally, we look at conventional and novel approaches for treating VRE infections, including antibiotics and combinations thereof, non-antimicrobial-based drugs, bacteriocins, bacteriophage therapy, probiotics, vaccines and the commensal gut microbiota itself.

#### Vancomycin resistance in enterococci

**Fig. 1:**
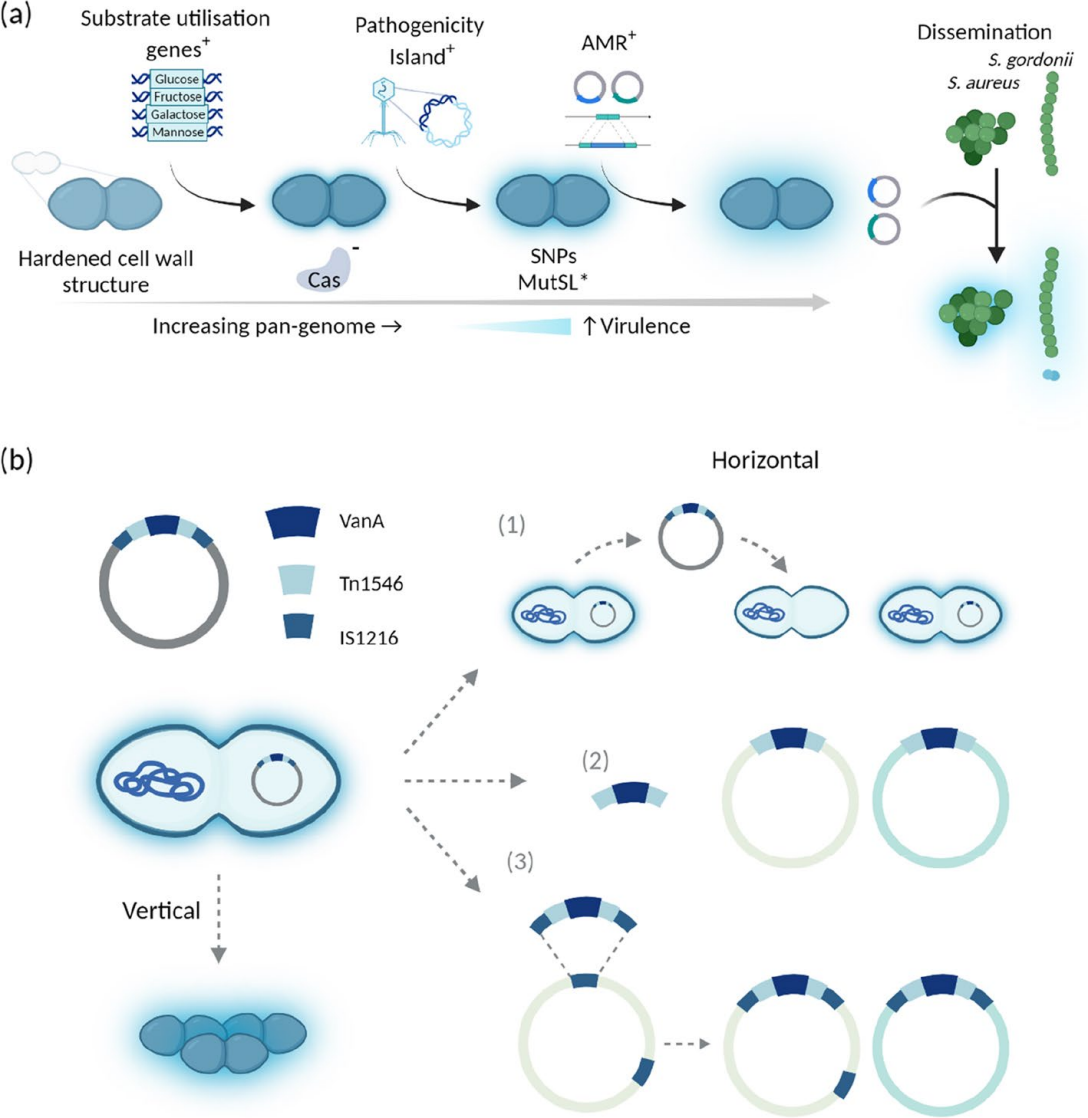
**a** Enterococcal acquisition of niche specific genes and (**b**) dissemination routes for *VanA* genes. **a** A change of niche resulted in *Enterococcus* spp. acquiring a harder cell wall structure and increased mutatable phenotype. *E. faecium* acquired various substrate utilisation genes, namely glucose, mannose, galactose and fructose. Hospital acquired infection-related *E. faecium* lack CRISPR-Cas systems, rendering them susceptible to receiving ectopic DNA resulting in the acquisition of pathogenicity islands, plasmids and insertion sequences. Hospital-acquired, hypermutable clade *E. faecium* can also have single nucleotide polymorphism (SNP) mediated resistance to antibiotics, such as fosfomycin, an antibiotic used to treat acute non-complicated urinary tract infections (UTIs). A hypermu table phenotype due to mutations in the DNA-mismatch repair proteins MutS and MutL increases the mutation frequency of strains. Enterococci act as genomic reservoirs for antimicrobial resistance (AMR) genes which can then be passed to recipients like *S. aureus* and *S. gordonii.* **b **The possible dissemination of VRE AMR genes due to transposons, insertion sequences (IS) and plasmids. Nested mobile genetic elements (MGEs) resemble the Russian doll model similar to carbapenemase resistance genes in *Enterobacteriaceae,* resulting in numerous horizontal dissemination routes including movement of the plasmid, transposition of the transposon between plasmids and homologous recombination [23]. Vertical dissemination occurs through daughter progeny containing the plasmid; this is confirmed by detecting the same plasmids and MGEs amongst the same clonal background. These are often responsible for hospital outbreaks accounting for ~ 30% of dissemination [23]. Horizontal dissemination: (1) Mobilisation of a plasmid to previously susceptible strains via conjugation ~ 7%. (2) Transposon-mediated mobilisation of sequences to other plasmids containing target sequences. (3) Mobilisation of IS. Most cases are caused by separate events indicating the high frequency that strains become pathogenic post-antibiotic treatment [23]. The notation “^+^” indicates acquisition of DNA, “*” indicates a mutation in DNA, “^−^“ indicates missing DNA feature

Enterococci possess intrinsic resistance to several groups of antibiotics, such as tobramycin, kanamycin, β-lactams and lincosamides (clindamycin, streptogramin) [21]. Due to their genome plasticity, enterococci quickly adapt to environmental changes [22]. HGT enables the acquisition of genetic elements that provide resistance to antibiotics and enable survival and persistence of enterococci in clinical settings (Fig. 1). *E. faecium* and *E. faecalis* represent two of the hardiest enterococcal species with capabilities to withstand multiple antibiotics, antiseptics, salt concentrations, organic compounds such as sorbic acid, and other stressors such as urea and high pH [4].

Vancomycin inhibits the formation of the cell wall by binding to the terminal D-Ala-D-Ala dipeptide of cell wall precursors, thus impeding processing into peptidoglycan [24]. Vancomycin bacteriostatic activity against *Enterococcus* is slow-acting, increasing the potential for the development of resistance [25]. Intrinsically resistant microorganisms possess a naturally different pentapeptide, such as in the case of *Lacticaseiobacillus paracasei*. Conversely, acquired resistance, as observed in *Enterococcus*, enables cells to synthesise modified cell wall precursors, for example, replacing the terminal D-ala of the dipeptide with D-lac or D-ser. This structural change results in up to 1000 times lower affinity for binding vancomycin [21].

Vancomycin resistance in enterococci was first described in 1988 [26]. *E. faecalis* was the predominant source of VRE; however, a gradual transition has occurred over the previous 20–30 years resulting in a species shift of VRE to *E. faecium,* although resistance is also detected in other enterococci [21, 27]. Vancomycin-resistance genes likely originate from un-sequenced soil bacterial species such as actinomycetes, the natural producers of glycopeptides [28–30]. Extensive genomic data from the human gut and skin microbiome suggests that the origin of *vanA* vancomycin resistance genes lay elsewhere and have moved by HGT, whereas *vanB* and *vanD* can be found in gut isolates, notably vanD protein orthologs in the gut commensals *Lachnospiraceae* and *Oscillospiraceae* [31, 32].

**Fig. 2:**
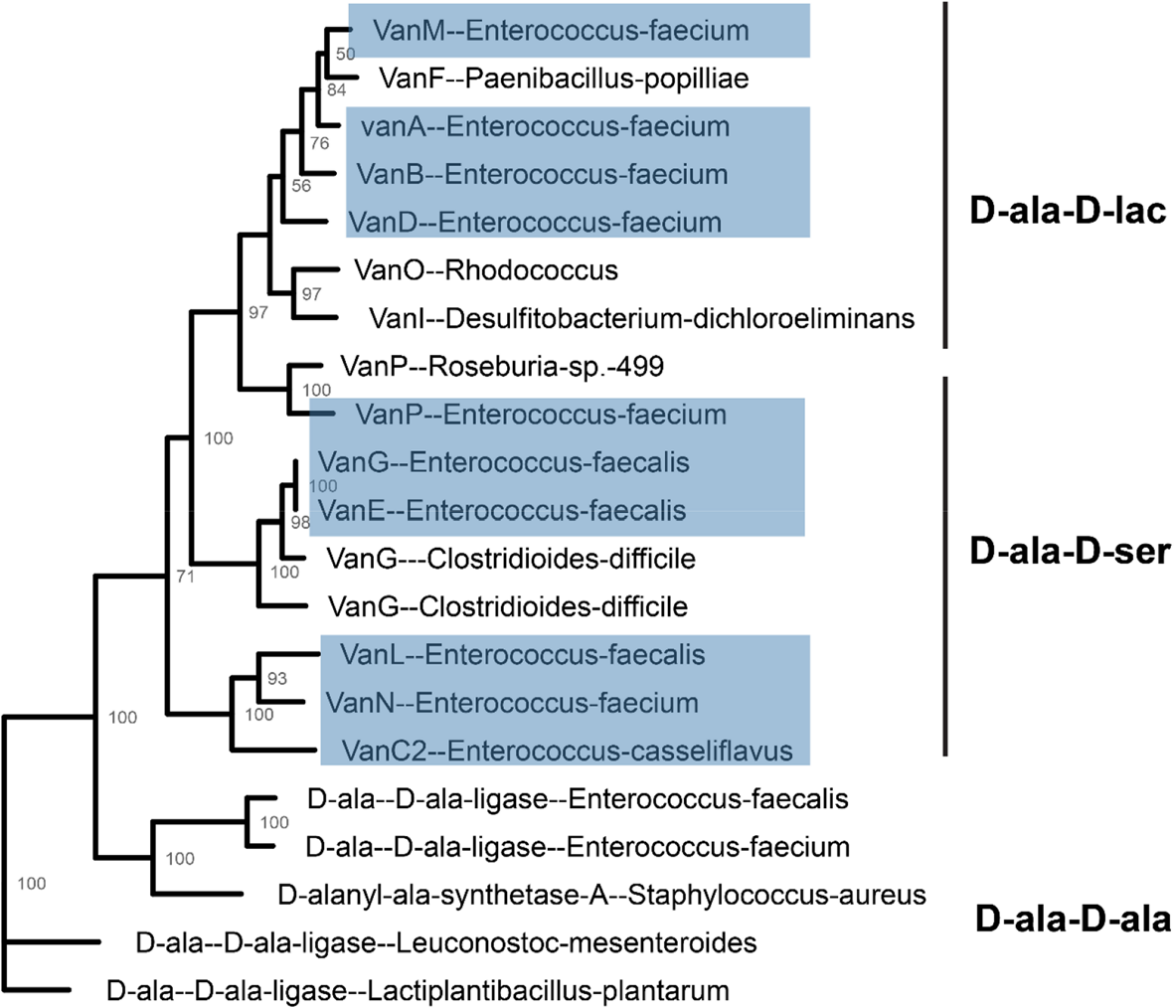
Phylogenetic tree of D-Ala-D-(X) ligases. Phylogenetic tree of ligases, those highlighted in blue are present among *Enterococcus*. The following accessions were used to construct the tree VanA [*Enterococcus faecium*] (AAA65956.1), VanD [*Enterococcus faecium*] (AAM09849.1), VanM [*Enterococcus faecium*] (ACL82961.1), VanC2 [*Enterococcus casseliflavus*] (AAA60990.1), VanE [*Enterococcus faecalis*] (ABA71731.1), VanL [*Enterococcus faecalis*] (ABX54687.1), VanN [*Enterococcus faecium*] (AEP40500.1), VanF [*Paenibacillus popilliae*] (WP_006285587.1), D-alanine–D-serine ligase VanG [*Clostridioides difficile*] (WP_021362548.1), D-alanine–D-serine ligase VanG [*Clostridioides difficile*] (WP_021425673.1), VanG [*Enterococcus faecalis*] (AAQ16273.1), D-alanine–D-alanine ligase [*Enterococcus faecalis*] (WP_002379157.1), D-alanine–D-alanine ligase [*Enterococcus faecium*] (WP_002293424.1), D-alanyl-alanine synthetase A [*Staphylococcus aureus *subsp.* aureus* str. JKD6008] (ADL66141.1), D-alanine–D-alanine ligase [*Leuconostoc mesenteroides *subsp.* mesenteroides* J18] (AET29676.1), D-ala D-ala ligase [*Lactiplantibacillus plantarum* subsp. *plantarum* ATCC 14917] (EFK27904.1), VanP [*Roseburia* sp. 499] (WP_075721811.1), VanP [*Enterococcus faecium*] (WP_222893641.1), VanI [*Desulfitobacterium dichloroeliminans*] (WP_041219811.1), VanO [*Rhodococcus*] (WP_209928075.1). The sequences were aligned using muscle [41] and the tree was constructed using RAxML-NG v1.2.0 [42] with 200 bootstrap replicates

In total, 12 types of vancomycin resistance mechanisms are known, 10 of which are described in enterococci (Fig. 2) [33–38]. Two major groups exist, categorised according to ligase activity, which is responsible for replacing the terminal D-ala with D-lac, referred to as D-lac ligases, or D-ser, referred to as D-ser ligases. The operons that encode D-lac ligases often result in high-level resistance with minimal inhibitory concentrations (MICs) > 256 μg/mL (*vanA, vanB, vanD and vanM, vanP, vanO, vanI*), while operons that encode D-ser ligases result in low-level resistance with MICs of 8–16 μg/mL (*vanC, vanE, vanG, vanL, vanN*) [21]. Strains of *E. gallinarum* and *E. casseliflavus* harbour the *vanC* operon on their chromosomes, contributing to low intrinsic resistance. *E. faecium* resistance is conferred by *vanA* or *vanB* operons, frequently carried on the transposable elements (TEs) Tn1546 and Tn1549, respectively [39]. HGT of vancomycin resistance has been confirmed among enterococci and other gram-positive bacteria, such as *S. aureus*, via plasmid transfer. This transfer of resistance is a significant problem, as vancomycin is a last-line antibiotic to treat the rising number of Methicillin-resistant Staphylococcus aureus (MRSA) infections [40].

#### Genomics and phylogenetics of disease-related enterococci

Although *E. faecalis* is the more common causative agent of enterococcal infections, *E. faecium* is more intrinsically resistant to antibiotics. Today, more than half of hospital enterococcal isolates in the US are resistant to ampicillin and vancomycin and have high-level resistance to aminoglycosides [22]. The ecological replacement of *E. faecalis* with *E. faecium* in the hospital environment could result from the intense use of antibiotics, the multiple antibiotic resistances of *E. faecium*, and the increased ability to withstand associated stressors [4, 43].

Genomics of pathogenic enterococci has shown characteristics that distinguish them from commensal isolates [44]. A review by Guzman Prieto et al. (2016) highlights the clonality of clinical enterococci [45]. A comprehensive study of 1644 *E. faecium* isolates by Arredondo-Alonso et al. (2020) identified the role of plasmids and their subsequent plasmid subpopulations amongst clinical VREfm and non-clinical sources [9]. Comparing isolates from hospitalized patients vs other sources, hospitalized patients carried a larger number of plasmids and plasmids were significantly larger in size. The plasmid population is the largest contributing factor to genome size outside of the core genome, and vast heterogeneity was observed among completed plasmid sequences with respect to plasmid length and number of replication and mobilisation proteins (*n* = 305). Commensal isolates have smaller genomes, while MDR isolates are promiscuous and have enlarged genomes that include plasmids, phages, insertion sequences (IS), and pathogenicity islands [44]. Enterococci act as an anchoring vector for these mobile genetic elements (MGEs), and up to 25% of enterococcal DNA can be accounted for by acquiring exogenous DNA through these mechanisms. A 2010 study identified that MDR isolates lack functional CRISPR systems [40], which enables MGE uptake. However, the largest study to date reported no difference between number of CRISPR-Cas systems and instead, found a type I restriction modification system (RM) enriched in clade A1 of *E. faecium* [9]. Separate RM S-subunits were enriched outside this clade suggesting it is the presence of RMs that dictate gene transfer events and drive subspecies separation [9, 46].

Fluctuation analysis of enterococcal genes described a favourable gain in niche-specific genes. Capturing MGEs and acquiring AMR genes in VRE has allowed gene-mediated survival within the hospital, where further MGEs can be acquired ad-hoc [47]. A recent analysis of the core genome of 973 global clade A1 (hospital associated) *E. faecium* isolates from 31 countries spanning 30 years defined 10 clusters. Low granularity was observed between groups, highlighted by core-genome admixture, which showed substantial ancestry between 78 isolates found at the boundaries, likely due to recombination events [17]. Similarly, a pan-genome analysis identified a significant number of shared genes among plasmids (40.9%), indicating plasmid-driven strain diversification among hospital clones. Low-frequency genes were also observed among plasmids across the pan-plasmidome, suggesting the acquisition of ectopic DNA to the accessory genome. Within the core genome, homologous housekeeping genes with > 5% divergence (*adk*, *atp*A and *pst*S) were observed, but high overall homology indicated clonal expansion of clade A1 [17].

A review by Hendrickx et al. (2013) summarises the role that enterococcal surface proteins play in the pathogenesis of *E. faecium* [48]. A large set of these proteins are anchored to the cell wall through a LPxTG domain and hence are exposed on the outside of the cell wall. These proteins can represent a pool of surface antigens for therapeutic exploitation that will be discussed later. Several virulence factors exist among enterococci, allowing persistence, evasion and competition among niche co-occupants. Haemolysin (cytolysin), a secreted toxin capable of lysing red and white blood cells, is often encoded in pheromone-responsive plasmids or pathogenicity islands and is associated with increased virulence among VREfs [49, 50]. Of note, the genetic capability to produce cytolysin was found in *E. faecium* via PCR but was determined to be a silent gene [51].

Gelatinase (*gelE*), found in > 90% of clinically associated clonal complex 17 (CC17) isolates, and other serine proteinases are responsible for degrading host tissues comprised of collagen to provide nutrients and can affect intestinal epithelial translocation [52, 53]. Gelatinase also modulates the host immune response and activates autolysin, which leads to the fratricidal release of extracellular DNA, a component in biofilm formation [54, 55]. *GelE* is found in both VREfm and vancomycin-resistant Enterococcus faecalis (VREfs) [56]. Hyaluronidase (*hyl*) degrades mucopolysaccharides of host connective tissue and extracellular matrix, enabling the spread of the cells and their toxins through host tissue whilst simultaneously providing a disaccharide carbon source. Although *hyl* genes are present among virulent enterococci, it is not a primary mediator of virulence. Aggregation substance (AS) promotes *E. faecalis* clumping and facilitates adhesion to eukaryotic cells, such as renal epithelial cells. It also mediates aggregate formation during conjugation and helps in high-frequency plasmid transfer and is not found in *E. faecium* [57]. *Esp* and *Esp*_*fm*_ genes are localised on pathogenicity islands within clinically relevant enterococcal species, where *Esp*_fm_ is a distinct marker for the hospital associated lineage CC17, and plays a role in adherence and biofilm formation among abiotic surfaces [58–60]. Microbial surface components recognising adhesive matrix molecules (MSCRAMMs) are essential in the early stages of infection. The cell wall-anchored enterococcal adhesins Ace and Acm are also present among clinically relevant *E. faecalis* and *E. faecium*, respectively. Transcriptionally expressed in the presence of urine, serum and collagen, and present as pseudogenes in non-clinical (NC) enterococcal spp., *Ace*-deleted mutants show reduced virulence for UTIs and endocarditis, highlighting their role in pathogenicity [50, 61]. Gls24, a general stress response protein from *E. faecalis,* is expressed in the presence of serum and urine, mediates bile salt resistance, and deletant mutants show reduced virulence [62]. Gls homologs are found in *E. faecium,* notably gls33 and gls20 [63]*.* NADH-peroxidase (*Npr*), alkyl hydroperoxide reductases (*Ahp*) and thiol peroxidases (*Tpx*) are three peroxidases responsible for reducing reactive oxygen species-mediated bacteriolytic activity of phagocytes among *E. faecalis* [50]. Interestingly, the roles of *Npr, Ahp and gpx* (a putative glutathione peroxidase) in *E. faecium* do not have a protective role against H_2_O_2_ [64]. Pilli also present virulence factors involved in biofilm formation, cell–cell aggregation and gene transfer, contributing to the pathogenesis of urinary infections and endocarditis and are present in both *E. faecium* and *E. faecalis* [50, 65].

#### Genomics of *E. faecium* suggest two distinct lineages exist

Initial molecular epidemiological analysis of *E. faecium* in the 1990s used pulsed-field gel electrophoresis (PFGE) typing, which revealed that a single clone dominated the enterococcal population in the USA [50]. Amplified-fragment length polymorphism (AFLP) was then used to differentiate the genetic relatedness of 255 isolates from hospitalised and non-hospitalised patients and animal sources [66]. From this host-associated genotypes were determined from AFLP fragment clustering, and showed, for the first time, host/environment associated ecotypes existed amongst strains of *E. faecium*.

Later, multi-locus sequence typing (MLST) analysis based on allelic differences in housekeeping genes defined lineages associated with hospital infections. Sequence types (ST) were determined and uploaded to an online database (https://pubmlst.org/efaecium/), which currently contains 2069 STs from 7593 isolates (18.7.2023). Based on ST, clonal complexes (CC) were defined, and in 2005 the term CC17 was defined representing a prevalent hospital adapted lineage of *E. faecium* present globally [67]. CC17 is associated with clinical strains, including ST17, ST117, ST78 and ST203 [50, 68]. Strains from the CC17 can persist in the nosocomial niche due to MDR, and biofilm formation, owing to the pathogenicity islands harbouring the *e*nterococcal *s*urface *p*rotein (*esp)* gene. This presents significant implications for catheter-mediated enterococcal invasive infection [33].

Several studies [33, 39, 69] have suggested the advantage of whole genome sequencing (WGS) in enterococcal phylogenomics compared to MLST, and have shown that recombination events across housekeeping genes are critical contributors to diminishing the usefulness of MLST applications resulting in a lack of accuracy and sensitivity.

The increasing number of enterococcal sequences has enabled comparative genome analysis, permitting scientists to elucidate differences between isolates originating from different sources and thus infer the evolution of strains [4, 11, 68]. On the other hand, a limitation of WGS lies within the downstream analysis, where analysis of the core-genome can overestimate the non-relatedness of isolates overlooking HGT events, resulting in missing forensic links of clinical relevance and ignoring the significance of the mobilome in the dissemination of resistance genes [23].

**Fig. 3:**
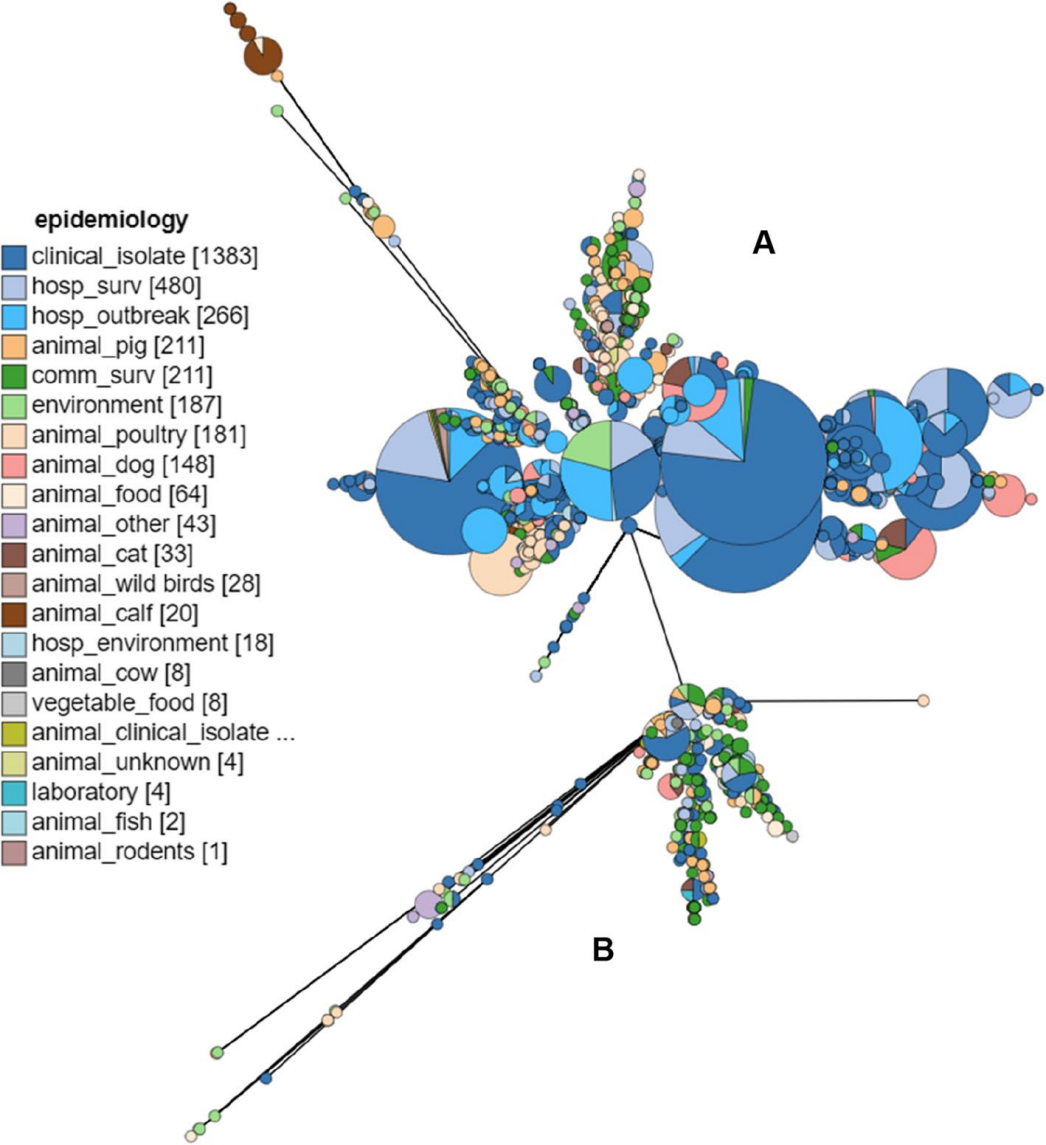
Separation of two distinct clades of *E. faecium*. This figure depicts concatenated genes of the cgMLST of 3308 isolates in a visual minimum spanning tree by GrapeTree on PubMLST.org [72]. The size of each node represents the number of isolates in a cluster, and each node is coloured with the epidemiological source of isolation using a pie chart. Only isolates with an epidemiological source were used to construct the tree. **A** represents clade A *E. faecium* and (**B**) represents clade B *E. faecium* / *E. lactis*

Comparative genome studies reported a general division of *E. faecium* into two separate clades, most often one encompassing community-related isolates and the second one including clinical isolates (Fig. 3). Core-genome phylogenomic analysis of 30 enterococcal strains belonging to four species (*E. faecium, E. faecalis, E. gallinarum *and* E. casseliflavus*) showed two distinct clades within *E. faecium* [68]. Based on average nucleotide identity (ANI) analysis, it was suggested that both hospital (A) and community (B) clades are potentially endogenous to the GIT of different hosts and now co-exist among human flora due to antibiotic elimination of competitors. Another option is that clades A and B diverge from each other due to antibiotic use and ecological isolation. Recombination between the two clades has been observed in the cases of two strains, representing a hybrid between clades A and B. A study by Been et al*.* (2013) identified recombination events between these two clades but found clade A to be notably more prone to recombination events, with the highest amount of recombination as a percentage of the core genome at 26.9% [70]. The source of recombinant sequences was also predicted to be vastly derived from clade B, confirmed by a recent study showing genomic admixture between clade A1, A2 and B [71].

The genes that contributed to the so-called “mosaicism” were acquired in a recombination event from clade B. In one strain, it was the occurrence of *adk-6* (adenosine-kinase) and *ddl-13* (d-Ala–d-Ala ligase) alleles and the presence of a CRISPR-Cas system, while in the second strain, it was the presence of the gene *pbp5* which can confer ampicillin resistance. Clade-specific traits exist; clade B isolates encode several secreted factors that can interact with eukaryotic cell surfaces, suggesting a closer association with host tissues in the GIT than clade A strains. Clade A strains may be more transient and associated with the GIT lumen, contributing to dissemination [68].

Another comparative genome analysis of *E. faecium* confirmed the existence of two phylogenetic groups [43]. The first group, community-associated strains (CA clade), did not carry AMR genes, certain genomic islands (GIs), or IS elements. The second group comprises hospital-associated isolates (HA clade) characterised by AMR genes and several unique IS elements, transposons, phages, plasmids, GIs, and polysaccharide synthesis loci 3 and 4. Genes encoding initiating transferase for polysaccharide biosynthesis and repeat unit polymerases are typically clustered in loci involved in polysaccharide synthesis within HA clade. Genomic analysis revealed a predicted polysaccharide-encoding gene cluster downstream of the enterococcal polysaccharide antigen (*epa)* region containing loci 1, 2 and 3 in all analysed strains. Loci 3 and 4 were primarily present in HA clade strains, while locus 2 and locus 1 were found in both clades not belonging to CC17. Inside Clade A, there was further evolution, and some of the clades share characteristics with community isolates, probably representing transitional lineages that lack IS and do not possess loci involved in *epa* extension. In addition, four of 21 analysed genomes in this study had the CRISPR-Cas locus; three were associated with the CA clade and lacked all antibiotic resistance. Some hybrid strains contained CRISPR and 8 antibiotic-resistance genes, representing another example of previously reported mosaicism. Clade HA specific characteristics most likely contributed to the emergence of this organism in the hospital environment in the last 30 years [43]. No CA clade isolates harboured any antibiotic resistance determinants. In contrast, all HA clade isolates have multiple resistance determinants, including the penicillin-binding protein 5 (*pbp5-R*) allele that confers ampicillin resistance, except for two strains [73]. Strain 1,231,501 in the HA-clade lacks all antibiotic resistance genes, including pbp5-R, but instead carries the replacement (hybrid) region, including *pbp5-S* which has an 8-fold decreased resistance. Strain E1039 has the *pbp5-R* allele but none of the other resistance genes and was genetically defined as an HA-clade isolate but came from a healthy volunteer, which explains the lack of antibiotic resistance determinants. Neither of these strains has IS16, which is considered an indicator of HA strains [73]. In the attempt to determine the evolution of *E. faecium*, this study suggested the existence of a primordial type of *E. faecium*, which split and evolved into two early community groups, with homologous *pbp5-S* or *pbp5-R* alleles, the latter representing community sources of ampicillin-resistant *E. faecium* [43]. These lineages could recombine with each other resulting in hybrid strains. The divergence between the two community groups reached core genomic differences creating two clades., HA and CA, which can be distinguished by *pbp5-R* (HA) and *pbp5-S* (CA) genotype. However, ampicillin resistant community derived isolates such as those from companion animals lie within this clade, suggesting it is also possible that ampicillin-resistant *E. faecium* clones evolved from animal reservoirs, or that animal ampicillin-resistant isolates represent evolutionary descendants of HA strains transferred to pets, a finding in accordance with a Dutch study of companion animals [9, 43, 74].

Analysis of differences among the two clades at the core genome level showed > 90% separated into two distinct groups, demonstrating that apart from the resistance genes and virulence factors (VF), essential differences in the core genes contributed to the differentiation of the two clades. The two clades separated between 1 and 3 million years ago, or approximately 300,000 years ago if accounting for a faster mutational rate. The proposed divergence occurred long before the modern antibiotic era [75]. These results contrast with a study by Lebreton et al. [11], which also determined the existence of two clades, A and B. Clade B comprised commensal strains. In contrast, clade A contains two sub-groups: clade A1 (epidemic hospital strains) and clade A2 (mixed animal and sporadic human infection isolates). The divergence of *E. faecium* occurred approximately 3000 years ago, coinciding with the development of housing, domestication of animals and specialised diets. The second event was the division of clade A ~ 75 years ago, which concurs with the introduction of antibiotics from a population dominated by animal strains. Hypermutable Clade A1 can acquire mobile elements and utilise carbohydrates of non-dietary origin. This study also identified hybrid clade A1 and B strains, confirming that human-infecting hospital and commensal strains occasionally overlap.

A study by Raven et al. (2016) showed that only two major lineages exist and did not support the existence of clade A2, which it found to be ancestral to clade A1, and whose phylogeny was consistent with a rapid clonal expansion of clade A from a single progenitor [76]. A recent genomic analysis of A and B clones encompassing 1128 genomes shows that clade A1 likely emerged as a clone from within A2, and due to ectopic genomic exchange that clade A2 HAI represents a continuum between clade B and clade A1, substantiating evidence for hybrid clones among the population in a direction towards clade A1 [71]. This is substantiated by the epidemiological origins of *E. faecium* strains in Fig. 3.

Further genomic analysis of 8,430 *Enterococcus* strains isolated from various sources, including abattoirs, retail meat facilities, animal sources, water sources and clinical isolates, revealed distinct clustering of enterococcal isolates to source, further suggesting specific adaptations to respective niches [77]. An analysis of ST between isolated sources showed ST117 was the most prevalent among VRE (76%) and non-VRE *E. faecium* (35%), suggesting clonal acquisition of vancomycin resistance elements. The frequency of AMR genes correlated to the isolation source. Vancomycin resistance genes (*vanA, vanB, vanC*,-type) were found solely in a clinical setting among *E. faecium*. Among the same strains from clinical sources, multiple other resistance genes were present (*dfr*G, *dfr*E, *msr*(C), *eat*(A), *aac*(6’), *tet*(LSM), *erm*(B), *aph*(6)-*la*). Genes *aac*(6’), *eat*(A), and *msr*(C) were found ubiquitously in the clinical setting, bovine faeces, feedlot catch basin, beef processing environment, natural water sources and urban wastewater. No vancomycin-resistant *E. faecium* was detected outside the clinical setting in this paper, which is contradictory to previous findings by other studies [11, 45, 78]. This highlights that VRE selection occurs within the clinical setting, driven by antibiotic exposure [77]. This data is backed up by a recent genomic analysis of Irish *E. faecium,* which does not cluster with isolates from the UK, its closest geographical neighbour, further suggesting independent events of acquisition and clonal expansion rather than dissemination of established VRE strains [79]. MDR *E. faecium* isolates were resistant to 4–7 drugs. The teicoplanin resistance gene *VanZ*, often co-localises with the *VanA* gene cluster and Tn1546, was detected in 46% of isolates [77]. Numerous VF were detected solely among clinical isolates and absent from other natural sources. Most virulence genes detected were thought to be ubiquitous among the species, involving traits which aid in colonising the GIT. These include capsular polysaccharide synthesis, biofilm formation and adherence, which are traits involved in the resistance to phagocytosis and bacteriophage infection. Cytolysin, a quorum-regulated cytotoxin, genes were detected among clinical isolates and rarely in natural water and animal sources, suggesting a lower-than-expected risk in the transmission of virulent *E. faecium* between animals and humans [77]. Clinical *E. faecium *isolates have an increased association with lysogenic phages, AMR genes, VF and MGEs. On average, genomes of clinical isolates contained 182 more genes than their NC counterpart, suggesting increased ectopic genomic exchange likely triggered by selective pressure [80].

Non-clinical (NC) isolates were enriched for genes associated with metal homeostasis and cadmium and copper resistance, likely linked to their addition to feed additives in the agricultural industry. Arredondo-Alonso et al. (2020) also found localisation of copper resistance operons to the plasmidome of *E. faecium* of porcine origin [9]. Cadmium and copper resistance were frequently observed within transposons of clinical isolates with *vanA*-type resistance in Ireland [79]. Within the former study, the localisation of virulence factors was enriched and localised to MGEs [80]. Copper resistance in *Enterococcus* spp. is attributed to the *tcrB* gene. High concentrations of copper sulfate in feed can co-select for glycopeptide resistance. Metal cation resistance genes can co-occur with AMR genes indicating a genetic linkage suggesting the potential for metals to drive AMR in human pathogens [81].

A recent suggestion has been made to re-classify the clade B *E. faecium* with *E. lactis* based on WGS data and an overall genome related index (OGRI) [82]. A pan-genome analysis of 181 genomes placed clade B strains amongst *E. lactis* using ANI where they were grouped with above 96%—the same species threshold. Subsequently, digital DNA-DNA hybridisation was used to discriminate between clade A1, clade B and *E. lactis* calculating genomic distances on a genome-to-genome basis which separated *E. lactis* and clade B from clade A using *E. lactis* type strain LMG 25958^ T^ as a reference [82]. Interestingly, this analysis highlights that multiple heterogeneous copies of the 16s rRNA gene can be present within the clade A with varying degrees of percentage identity to the *E. lactis* reference. A similar ANI value was determined amongst clade B and clade A *E. faecium* and high rates of recombination and IS elements were suggested to be responsible for bifurcation of the clade in 2007 [68, 83].

Gene associations of NC and CL isolates show a clade specific gene content, these are supported by gene flux studies, where CL isolates show adaptation to the hospital. In contrast, commensal or NC contain genes associated with glycerol fermentation to the biosynthesis of 1,3-propanediol, among others [4]. Global dissemination of clade A1 *E. faecium* contains blurred edges within the clade partly due to mobilised elements such as plasmids and phages, but also due to recombination events within the core genome, indicating evidence of clonal expansion and dissemination may be distorted due to these events complicating genomic analysis due to enterococcal genome plasticity [11, 17, 76]. Not all aetiological concerns remain within clade A1. The discovery of an active botulinum neurotoxin-like toxin within a commensal *E. faecium* on a conjugative plasmid (pBoNT/En) may present a significant risk to public health if recombination events occur between MDR pathogenic and a toxin-containing commensal strain, highlighting the need to monitor the NC clade [84].

#### Genomics of *E. faecalis* reveals lower diversity among strains

*E. faecalis* was previously responsible for over 50% of hospital-associated enterococcal infections [1]. Similar to *E. faecium*, STs exist for *E. faecalis*. Currently, the MLST database contains 2942 isolates consisting of 1470 STs (https://pubmlst.org/efaecalis/ accessed on 21/7/23), with strains from ST6, ST9, ST21 and ST40 often linked with hospital infections [68].

Among 18 analysed *E. faecalis* genomes, low phylogenetic diversity was observed, and most diversity can be linked to the increase of MGEs, mainly prophages, conjugative plasmids and transposons, while the core genome seems highly conserved. The bigger genome sizes were characteristic of strains lacking CRISPR-Cas systems [68]. In contrast to *E. faecium*, a multiclade structure was not mirrored in *E. faecalis*, for which the acquisition of mobile elements also drives diversity. Antibiotic resistance and pathogenicity island traits have converged in *E. faecalis* lineages. Despite the convergence of similar features in those lineages, substantial differences in genome sizes (2.74 – 3.36 Mb) and gene content exist where some *E. faecalis* strains only share > 70% of gene content. Still, high homology exists within similar genes (> 99% ANI) [68]. Where genome size differed, increased size was related to compromised genome defence due to a characteristic lack of CRISPR-Cas systems. Increased distribution of MGEs domains, plasmid mobilisation MobC (PF05713), anti-restriction protein ArdA (PF07275) and transposase domains (PF01526) exist more frequently among genomes > 3 Mb [68]. Ecotypes defined by specific MGE may be identified within high-risk lineages or in lineages with variable CRISPR-Cas status (e.g., ST40 and ST21) [68].

A second comparative analysis of 38 NC and CL *E. faecalis* isolates showed little differences in genome size (~ 3 Mb), number of coding sequences (CDS), presence of MGEs, VF and AMR genes [80]. Hierarchical clustering of 8032 *E. faecalis* orthologs identified for 38 genomes showed no evidence of distinct lineages. The genomes of NC and CL *E. faecalis* isolates lacked specific structural and functional features, and clade separation based on ortholog presence/absence between NC and CL strains did not apply. These differences indicate that *E. faecalis* strains examined to date constitute a single lineage specifically adapted to the GIT and subjected to genome expansion. Such distinctions may be the cause of the earlier appearance of antibiotic-resistant strains of *E. faecalis* than of *E. faecium* [80].

A more recent comparative genome study on 78 strains of *E. faecalis* from the GIT, faeces, blood, urine, water and dairy products was conducted. Still, no direct link between isolation niche and phylogenetics was confirmed. However, some environment-specific genes were found, and blood isolates harboured the highest number of antibiotic-resistance genes where *vanA* and *vanB* gene clusters were found [85]. Analysed isolates harboured 116 intact prophages and no correlation of source to the number of prophages was observed.

Another study that compared 168 genomes isolated from the UK and Ireland, including 58 VREfs strains and 110 VSEfs strains, to the core genome *of E. faecalis* V583 revealed 124,194 single nucleotide polymorphisms (SNPs) over 2,886,189 nucleotides. The SNP-based phylogenetic tree showed that 53% of strains clustered into three distinct lineages (L1, L2 and L3). Isolates in L1 belonged to ST6, ST384 and ST642, all part of CC2, and L2 isolates were ST28 and ST640, belonging to CC87. Both of these clonal complexes are associated with hospital isolates. Isolates in L3 were identified as ST103 (CC388), previously reported in a limited number of clinical isolates. Comparing these isolates with 347 globally isolated *E. faecalis* strains, the phylogenetic tree based on 1293 genes showed that isolates belonging to L1 and L3 isolated in the UK and USA were genetically distinct, suggesting independent clonal expansion of the lineages with limited international dissemination [76].

An analysis of 2027 *E. faecalis* isolates using both long and short read sequencing from sources including clinical, non-clinical, avian and farm animal isolates by Pöntinen et al. (2021) determined that the emergence of hospital associated lineages pre-dated modern-day hospitals [10]. Molecular dating places ST6 in the mid 1800s. The adaptation of the generalist *E. faecalis* to the hospital setting is a product of its evolution in a broader set of niches. This aligns with a previous study by Lebreton et al. (2017) [4]. The ability of *E. faecalis* to persist in different niches is due to its generalist nature in comparison to the genomically plastic *E. faecium*, which is more promiscuous, hence its adaptive evolution through HGT has favoured its persistence in the clinical setting.

#### Genetic insight into vancomycin resistance

Globally, the distribution of resistance mechanisms is mixed, and geographical location seems to drive genotypic mechanisms of resistance. Countries tend to be populated by either of the two mechanisms, rarely both. This suggests potential competition between existing clones where both mechanisms exist, isolated regions of selective pressure or clones arising with both mechanisms [17, 76, 86]. The *vanA* operon is predominant in the US, Ireland, and central Europe. In contrast, *vanB* appears to be dominant in Australia and Germany [17, 39], but this relationship seems fluid, with *vanA* starting to increase in Australia [87]. In vancomycin-resistant isolates of *E. faecium* isolated in Australia, *vanB* was located predominantly on the chromosome, but in three isolates, Tn1549 carrying plasmids were detected [39]. Transposons of the same structure tended to insert at the same chromosomal location, but it is not a strict rule. Vancomycin-resistant isolates were scattered across branches of clonal phylogeny, implying both transposon acquisition and loss events. All detected transposon gain events occurred in isolates related to or identical to vancomycin susceptible *E. faecium* (VSEfm) isolates, consistent with the suggestion that VRE emerges from the circulating enterococcal population followed by clonal expansion and VRE transmission. Patterns of genomic diversity from different hospitals did not differ but seem to have arisen from a common ancestor [39]. The molecular characterisation of VREfm and VREfs isolates from a Chinese hospital confirmed that all 76 isolates harboured *vanA* resistance [88]. On the other hand, a study by Chen et al. (2015) showed that *vanM* gene was more dominant compared to *vanA* among 70 isolates from hospitals in Shanghai, China. The presence of vancomycin resistance genes among NC isolates differs among studies. A study of VRE from animal sources in Korea identified 44% and 17% of isolates from poultry meat and poultry faeces, respectively, containing a *vanA* gene, but the analysis was not at WGS resolution [78]. Similarly, *vanA* positive enterococci were detected among domesticated animals [89].

The majority of 250 VREfm isolates from patients confirmed as having bacteraemia carried the *vanA* resistance gene [76]. All but one VREfm belonged to hospital associated clade A. One clade B (community associated) isolate was *vanA* positive, showing that vancomycin resistance is not restricted to the hospital associated lineage. The transposon encoding *vanA* was predominantly located on plasmids, while *vanB* transposon was predominantly inserted into the chromosome [39, 76]. *VanB*-type transposons were inserted at identical sites in the chromosome, suggesting acquisition followed by clonal expansion. Eleven VSEfm isolates carried *vanA* or *vanB*. In the case of *vanB*, these sensitive isolates lacked regulatory genes. Still, the presence of bacteriocin genes on the associated transposon may explain their retention in the genome [76]. Similarly, Howden et al. (2013) showed that VSE isolates were genetically indistinguishable from *vanB*-carrying isolates. Another comparison of 200 VREfm and 93 VSEfm concluded that they often belong to the same cluster in the phylogenetic tree. Genetic relatedness of transposons carrying the *vanA* gene revealed that 4 of 6 phylogenetic clusters contained more than one transposon type (based on deletions and/or SNPs), suggestive of de novo acquisitions of vancomycin resistance by hospital VSE strains [69]. This corresponds to the findings showing the same trend of repeatedly introducing vancomycin resistance into the VSE hospital population [79, 90].

The genetic basis for vancomycin resistance among 168 VREfs and VSEfs strains isolated in the UK and Ireland was investigated [91]. In total, 99% of VREfs strains carried *vanA* resistance genes. All three dominant lineages reported above (L1, L2 and L3) contained mixtures of sensitive and resistant genotypes. However, variation in gene content was considerable; transposase, resolvase, *vanY* and *vanZ* were not detected within some isolates, but vancomycin MIC values remained > 256 mg/L suggesting genomic rearrangement but a sustained phenotype. Additionally, since the major VREfs lineages were also the common lineages for VSEfs, the control of VREfs is likely to depend on defining and addressing drivers for VSEfs and their transmission [91].

Transference of vancomycin resistance among commensal *E. faecium* (clade B) is possible [92]. Two *E. faecium* strains isolated from an immunocompromised patient in France, one from blood (UCN71) and one from faeces (UCN72) with commensal clade B origin, were described as possessing *vanN* type resistance. Only two more examples of strains carrying *vanN* resistance have been reported, and these were isolated from Japan (clade B) and Canada (clade A). The *vanN* determinants were almost identical between France and Japan but entirely different from Canada, suggesting independent acquisition events. In all cases, the *vanN* determinant is localised on a conjugative plasmid. Analysis of SNPs on the two isolates from an immunocompromised patient in France identified two non-synonymous SNPs within the *VanS* gene, which encodes the regulatory system controlling the expression of resistance [92].

Finally, a GI associated with *vanD* type of resistance in six VREfm Dutch isolates was identified [93]. Phylogenetic analysis of *vanD* gene clusters from the six Dutch isolates and 13 *vanD* gene clusters retrieved from GenBank, showed that Dutch isolates did not form a single branch and that *vanD* gene clusters did not group according to the species in which they were present. The six Dutch strains harbouring *vanD* were not epidemiologically related. This lack of evidence of clonal spreading suggests that *vanD* VREs are not transmitted between patients, unlike *vanA* and *vanB* strains. A considerable similarity between GI carrying *vanD* in anaerobic gut bacteria and in six *vanD E. faecium* in this study supports the idea anaerobes could be a source of *vanD* type of resistance, but this requires further experimental confirmation [93]. Recently, the first plasmid-borne *vanD1* gene cluster was identified in *E. faecium* (MIC, 16 μg/mL). The strain is constitutively vancomycin resistant, due to deleted *vanRS*, has a presumed inactive native *Ddl* ligase, due to frameshift mutation and contains the vancomycin resistance cluster on a highly conjugative plasmid of 10^−4^ to 10^−5^ per donor cell [94].

#### Rinse and repeat; transmission, recurrence and epidemiology assessed by WGS

The use of vancomycin, cephalosporins and quinolones selects for pools of VRE within patients [95, 96]. Ceftriaxone, a third-generation cephalosporin antibiotic that causes extensive perturbation of the gut flora, has been associated with VRE proliferation, likely because of the collateral damage on the microbiota and has been associated with increased bloodstream infection incidence [96]. Another study identified ceftriaxone use as a risk factor for *C. difficile* infection [97].

The first study that used a WGS approach in a clinical application developed a core genome MLST (cgMLST) to standardise isolate comparisons [98]. This approach identified 1,423 cgMLST target genes. In an analysis of 103 outbreak isolates from five different hospitals, the cgMLST successfully distinguished epidemiologically related isolates, even between sequence types (ST). WGS found its application in elucidating the transmission of VREfm [69]. When 293 *E. faecium* genomes were analysed, 291 were hospital associated, while only two belonged to clade B, although both were healthcare-associated. A total of 284 genomes formed a highly related clonal expansion within the hospital clade, and more than half of the isolates were highly related to at least one other isolate. After a maximum likelihood tree was constructed based on SNPs, numerous clusters of highly related isolates were detected, suggesting multiple introductions of *E. faecium* into the hospital, followed by clonal expansion, transmission, and persistence [69].

A WGS study of *E. faecium* from Ireland, a country with high invasive *E. faecium* prevalence, revealed that most Irish isolates cluster outside global isolates, where ST80 *vanA* resistance was the predominant subtype. This subtype generally contained < 4 allelic differences suggesting inter-hospital transmission [79]. Dissemination of *vanA* by TEs between linear and circular plasmids was highlighted. A hybrid plasmid was sequenced during conjugation experiments highlighting the promiscuity of plasmid-plasmid interaction and substantiating evidence of mosaicism among the plasmidome of VREfm observed in other studies [23]. The paper also reported the first evidence of *E. faecium* with multiple insertions of IS1216E. The authors suggest that numerous copies of the IS could lead to further genomic instability and aid in dissemination, in particular in the absence of transposable elements TEs, which may be indicative of such a high prevalence of *E. faecium* among Irish hospitals [79]. This paper also highlighted TEs with mutations and deletions resulting in truncated TEs, some non-typeable *vanA* resistance isolates had > 50% of TE truncated, indicating IS mediated dissemination of *vanA* genes, potentially via a “copy-in” mechanism, suggested by the presence of multiple IS elements [79].

A recent analysis of the dissemination of *vanA*-type VRE among Dutch hospitals identified clonal spread, plasmid dissemination, Tn1546 mobilisation and mixes of both for causative genomic dissemination (Fig. 1) [23]. Clonal dissemination was responsible for ~ 32% of the spread, and ~ 59% of cases were unrelated, suggesting repeat independent introduction of resistance to hospitals. Similarly, a multi-jurisdictional outbreak of VREfm studied in Japan concluded clonal dissemination facilitated spread between hospitals and that current containment measures were insufficient [99]. WGS analyses of *E. faecium* between UK and Ireland, Denmark and Australia further highlighted the potential for clinically relevant clones to disseminate between hospitals [76, 90, 100]. Dissemination of these clones could partly be due to carriers, where it is estimated for every VRE patient, there are 2–10 fold more transient carriers [101].

A study quantified acquisition rates among patients for *E. faecium* using a longitudinal, sequence-driven method of genomic surveillance. Half of all environmental swabs (*n* = 922) were positive for VREfm, and the majority (60%) of clade A1 positive patients had genomic links to other patients or environmental swabs such as medical devices and communal areas [102]. Polymicrobial colonisation of *E. faecium* among patients existed, and invasive infection due to patients’ gut-colonising strains occurred, highlighting the interface for potential HGT events and the ability of strains to become pathogenic.

Studies on lower numbers of strains also reveal interesting relations among VRE strains. In the comparative genome analysis of four VREfm strains isolated from two fatal cases (VRE2 from patient X and VREr5,6 and 7 from patient Y), VREr6 and VREr7 were approximately 140 kb larger, due to the presence of plasmids and phages. Phylogenomic analysis suggested that patient Y was infected by VRE strains from more than one lineage. While Qin et al*.* (2012) showed that clinical isolates of *E. faecium* do not carry CRISPR, in this study, CRISPR-like regions were identified in all four isolates, but without active *cas* genes, and thus with no functional significance. In addition, the majority of common virulence factors were encoded in the four genomes and all of these strains showed multiple resistance profiles, but were sensitive to linezolid [33].

Genomics can also assess relapse and reinfection of *E. faecium* bacteraemia. Recurrent bacteraemia by infection with genetically different strains is a driver for relapse VRE cases. This is potentially attributed to polyclonal populations among patients, carrier events introducing new strains or environmental transmission. The timeframes of infection relapse due to the same or a novel strain did not differ (within 108 days), although the sample size was limited (*n* = 21). This study also showed that mixed infections commonly originate from an intravascular source, suggesting that central venous catheters are colonised with multiple *E. faecium* strains. Asymptomatic carriage and environmental sources contribute to the circulating pool of VREfm acting as potential contamination sources, likely attributed to the ability to withstand common disinfectants and handwash alcohols [4, 103, 104].

A study of 80 VREfm in Australian hospitals from patients with and without clinical symptoms of VREfm infection, incorporating the spatio-temporal location of patients, also highlighted dissemination routes [87]. The intensive care unit was the most likely location of VREfm transmission, followed by the acute general medicine ward. Most VREfm were isolated from asymptomatic patients, who represent reservoirs of VREfm and are the starting point of “silent” dissemination of VREfm to high-risk patients, healthcare workers and the environment. This study showed the merit of examining isolates from asymptomatic colonised patients in understanding VREfm transmission routes in hospital environments [87]. A recent Danish study suggests routine screening of recurrent patients upon re-admission to circumvent inter-hospital carrier events could help both clonal dissemination and potential HGT events, but only employed on a cost–benefit basis among high prevalence hospitals [105].

Hospitals also represent a putative source of environmental contamination, as hospital-adapted lineages can be found in wastewater plants not restricted to plants in geographical proximity to hospitals [87]. The subsequent genomic analysis identified genetic intermixing between clinical and wastewater isolates of *E. faecium* at sites which did not process hospital wastewater, suggesting the spread of clinically relevant isolates among the community affecting the enterococcal gene pool [106]. Treatment plants did not knowingly receive farm effluent suggesting animal-derived lineages are carried among community members. On the contrary, Canadian genomic surveillance of enterococcal spp. on a One Health basis did not identify community VREfm beyond wastewater reservoirs but did find VREfs among beef processing facilities [77]. Natural water and feedlot catch basins were free from detectable VRE, but vancomycin-resistant *E. hirae* was detected in urban wastewater. Phylogenomic relatedness between animal-associated and clinical clades suggests little transmission through these routes, i.e. minimal bovine-associated dissemination, and urban wastewater contamination is likely a result of human carriage [77].

**Fig. 4:**
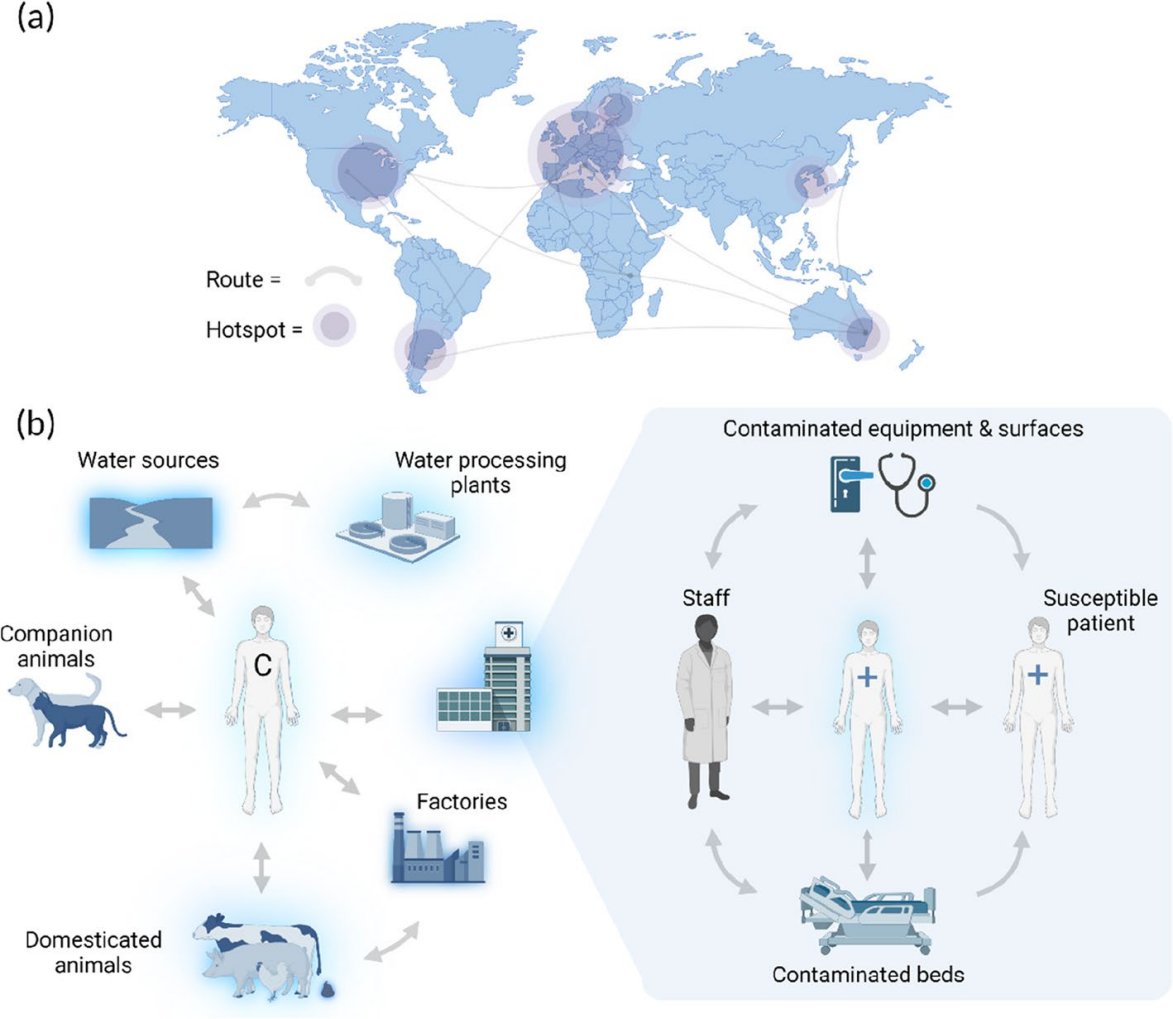
Epidemiological spread of VRE. This depicts the (**a**) global [17], (**b**) community [77] and (**c**) clinical routes of dissemination of VREfm [27, 50]

An overview of the routes of dissemination is found in Fig. 4. Global dissemination patterns of *E. faecium* highlight levels of global dissemination among clusters followed by local adaptation and regional dissemination [17]. Generally, clonal dissemination patterns can be traced by core-genome signatures. However, European homogenisation patterns indicated by significant recombination events across clones suggest the co-circulation of multiple subclones that augment the European VRE gene pool and potentially lead to new lineages.

#### The role of genomics in epidemiology, detection, prevention and treatment

Routine WGS is common practice when processing samples from hospitalised patients with VRE. This practice has been implemented globally to track outbreaks, epidemiological routes of transmission and decipher characteristics of clinically relevant sequence types [17, 23, 60, 79, 86–88, 91, 106–109].

Diagnostics, resistance profiling, outbreak tracking, personalised medicine, epidemiological tracking of clinical strains, identification of new CC/ST and the monitoring of surfaces by sequencing are some applications in the detection and control of VRE. A review by Rogers et al. (2021) explores the role of WGS in surveillance of enterococcal species and concludes that WGS and a One-Health surveillance approach play a key part in tracking enterococcal species [110]. A recent study implemented a surveillance programme with an infection prevention and control nurse specialist to use genomic insights into the management of AMR spread within the healthcare system [111]. The usefulness of WGS is hinged on the ability to sequence, interpret, and implement the findings in a timely manner. The study had a median turn-around time of 33 days from sample sequencing to final report – but in some cases reports were generated within 10 days [111]. Although time consuming and likely resource expensive, it has previously been shown that real-lime metagenomics is a viable detection tool for the identification of bacterial pathogens [112, 113].

#### Current chemotherapeutic treatment of VRE infections

VRE are causative agents of infective endocarditis, catheter-related bloodstream infections, urinary tract infections, bacteraemia, abdominal and pelvic infections, and less often CNS and skin infections [22]. Genomic surveillance is not enough to deter expansion of VRE in the clinical setting and cleaning practices and hygiene remain paramount in preventing dissemination [114]. An extensive recent review by Cairns et al. (2023) captures the limited array of treatment options available for VRE blood stream infections, the complexities of dosing regimens and promise of therapeutic drug monitoring (TDM) for improving clinical outcomes [115]. In the USA, the only agents approved to treat VRE infections are quinupristin-dalfopristin and linezolid but both display serious side effects, which limit their application in treatment. Quinupristin-dalfopristin is a streptogramin antibiotic active only against *E. faecium* [22]. On the other hand, linezolid is a bacteriostatic agent and, therefore, not appropriate in cases of severe VRE infections, such as endocarditis. A recent review by Turner et al. (2021) highlights the expanding discovery of transferable linezolid resistance among VRE and *Staphylococcus* spp. [116]. A study of 154 linezolid resistant isolates from Ireland showed 22.7% of isolates had genes capable of transferable linezolid resistance, with 8 enterococcal species containing a plasmid bound determinate, *optrA* (oxazolidinone phenicol transferable resistance)*,* 19 isolates harboured *poxtA* (phenicol oxazolidinone and tetracyclines) within an IS element [117]. These determinants have also been found in Tunisia and China [118–120].

Daptomycin is a cyclic lipopeptide capable of penetrating biofilms, that disrupts the integrity of the membrane and has increased potency compared to other antibiotics [121]. The peptide is calcium-dependent and targets cell wall biosynthesis, acting like a cationic antimicrobial peptide [122]. Detailed analysis of the activity of daptomycin in the treatment of VRE can be found in Munita et al. [123]. The optimal dosage prescription varies depending on VRE status, and an updated dosage of 8–12 mg/kg q24h and 6 mg/kg q24h for *E. faecium* and other enterococcal spp. respectively, with a high-dose daptomycin (> = 10 mg/kg) preferential due to improve clinical outcome [115, 124]. A recent review by Miller et al. (2020) explores the development of resistance to the newest anti-VRE compounds [120]. Additionally, there have been reports of the development of resistance to daptomycin and off-target mutants [18, 125].

A single-centre, retrospective cohort study of 93 adult inpatients with VRE bacteraemia treated with either linezolid or daptomycin suggested that daptomycin at standard doses (6 mg/kg) is associated with a higher rate of clinical failure relative to linezolid [126]. In spite of the concern of side effects of bone marrow suppression, linezolid-treated patients had a relatively low mortality rate even with a majority of patients possessing an underlying immunocompromising condition [126].

Four meta-analysis studies that compared linezolid and daptomycin treatments in bacteraemia showed that daptomycin was connected with higher mortality levels compared to linezolid. The first observational retrospective study, conducted in 2013, showed that there may be a difference in favour of linezolid, since it was associated with increased survival (*p* = 0.053). Although sample sizes remained small - (*n*=54–235), no statistical significance was observed, majority of studies described dosing at 6 mg/kg, and the study was not adjusted for confounders [127]. Another retrospective study involved 13 studies which fulfilled conditions (clinical trials or observational studies of the treatment of VRE bacteraemia that reported daptomycin and linezolid treatment outcomes simultaneously) showed that daptomycin use was not associated with better microbiological cure, but the mortality levels were significantly higher compared to linezolid treated patients, but sample size remained small [33–200]. In another analysis of 10 studies providing mortality data on patients older than 18 years, patients with VRE bacteraemia treated with daptomycin had a significantly higher 30-day all-cause mortality and infection-related mortality. Relapses were also higher, suggesting that daptomycin may be associated with worse outcomes in patients for VRE bacteraemia compared to linezolid [129]. A fourth study found an overall significantly higher rate of treatment failure for daptomycin at standard dose (6 mg/kg) compared to linezolid treatment [130].

Contrasting results were reported in a larger, more methodologically robust study. A national retrospective cohort study among patients between 2004–2013 based on 30-day all-cause mortality, microbiological failure, and VRE recurrence in blood showed that linezolid was associated with a significantly higher risk of treatment failure and mortality, but no difference in recurrence was observed [131]. Daptomycin was better in this cohort study even though the dosage was lower, and higher dosages are thought to improve clinical outcomes from bloodstream infections. This study makes the stronger case compared to previous meta-studies as it was performed on carefully selected patients, who were receiving a single medicine treatment, and outcome measurements were pre-defined for clinical relevance, although some limitations are included i.e. 97% of male patients and relatively low number of transplantation patients, who are often susceptible to VRE infections [125]. A subsequent follow-up study analysed sequential therapy vs continuous therapy, as the former cohort had been omitted from the initial study and reported that patients switching to daptomycin associated with lower mortality and prolonged time of the switch was correlated with increased mortality [132]. A more comprehensive review of daptomycin-linezolid meta-analysis suggests a randomised controlled trial of high dose and low dose daptomycin is required, but current data highlights the value of daptomycin as a treatment of VRE bacteraemia which aligns with the extensive review by Cairns et al. (2023) [115, 133]. Increased dosage, combinatory therapies and TDM offer possible ways to enhance daptomycin efficacy [115]. The synergy between daptomycin and ceftriaxone was assessed in the simulated endocardia vegetation model, and the combination was more active compared to daptomycin alone, with enhancement associated with a reduction in cell surface charge [134]. An in vitro pharmacokinetic/pharmacodynamics model was used to assess whether beta-lactams are able to enhance daptomycin activity against VRE and reported that ceftaroline and ertapenem were able to synergise with daptomycin against 2 VREfm and a VREfs to prevent the emergence of daptomycin non-susceptibility [135]. Similarly, fosfomycin-containing regimens delayed the emergence of daptomycin non-susceptibility in 2/3 strains tested, while an increase in MIC was observed when daptomycin was used alone [136]. However, recently it was shown that resistance to fosfomycin emerges rapidly. Until now, *fosB*, which catalyses the Mg-dependent addition of L-cysteine to the epoxide of fosfomycin, was the only known plasmid-borne resistance determinant in *Enterococcus* spp. The plasmid-encoded *fosb* gene inserted into the vanA type transposon is responsible for the high fosfomycin resistance in VRE. Recently, the complete sequence of a novel *E. faecium* plasmid containing two copies of *fosB* was obtained. The co-existence of *vanA* and *fosB* in the same conjugative plasmid was confirmed [137]. Although costly, the implementation of real-time metagenomics and sequence-based tailored regimens may keep current therapeutic interventions viable, amidst the rising cases of resistance, if implemented on a case-by-case basis.

Tigecycline is a recently developed glycylcycline antibiotic, from the tetracycline family of antibiotics, with broad spectrum activity against VRE, MRSA, *C. difficile* and gram-negative pathogens including *Acinetobacter baumannii, Klebsiella pneumoniae,* and *Escherichia coli* [138]. The antibiotic is used as a last resort to treat complicated skin and intra-abdominal infections and has a low MIC (0.03–0.5 µg/mL) against VRE but resistance to tigecycline has been documented since its first clinical application [138, 139]. A recent meta-analysis of the prevalence of tigecycline resistance among VRE highlighted a 1% and 0.3% prevalence of resistance globally among *E. faecium* and *E. faecalis*, respectively, with a 3.9% resistance for *E. faecium* in Europe [140]. The authors suggest that the prevalence of resistance may be higher globally, and more in line with Europe, due to reduced routine microbial susceptibility testing programs which are not a global standard. A mechanism by which *E. faecium* and *E. faecalis* can acquire resistance involves the mutation of RpsJ, a ribosomal S10 protein of the S30 subunit – which is in close proximity to the binding pocket of tigecycline resulting in reduced affinity [141]. Transferable tigecyline resistance among *E. faecium* has been observed which involves upregulation of plasmid-bound *tet*(L) and *tet*(M) genes encoding a major facilitator superfamily (MFS) efflux pump and ribosomal protection protein, respectively [142]. High-level resistance determinants (MIC value of 32-64μg/mL) *tet*(X3) and *tet*(X4) encoding proteins which catalyse tetracyclines rendering them inactive have been found also on plasmids from *Enterobacteriaceae* and *Acinetobacter*, but not from clinical sources [143]. The detection of these resistance determinants coincides with the use of tetracycline antibiotics as growth promoters in animal production or metaphylaxis treatment – which mirrors the use of avoparcin and the rise of VRE [143]. Notably, these high-level, plasmid-bound determinants have not been found in VRE to date.

#### Non-chemotherapeutic anti-VRE alternatives

##### Bacteriocins

Bacteriocins are ribosomally-synthesised antimicrobial peptides with highly specific or broad antimicrobial ranges, often against related strains. They are produced by both gram-positive and gram-negative bacteria, and it has been stated that 99% of bacteria produce at least one bacteriocin (Klaenhammer, 1988). They have gathered much traction as an alternative to antibiotics due to their reduced collateral damage on the microbiome, and can be broadly categorised into two groups, the highly diverse modified class I bacteriocins (< 50 kDa) and unmodified (< 10 kDa) class II bacteriocins [148–150]. Recent tools, such as BAGEL4 and Antismash7 have mined the vast genomic data available to find novel bacteriocin gene clusters and expanded the repertoire of putative gene clusters [151–153].

Bacteriocins have diverse mechanisms of action, including pore formation, subsequent cell leakage and lysis, degradation of cell wall peptidoglycan or interference with cellular processes such as transcription, translation and DNA replication [154, 155]. Due to their safety and high specificity, bacteriocins represent promising alternatives in the fight against multidrug-resistant pathogens, including VRE. However, only a limited number of studies have investigated the application of bacteriocins against VRE and multidrug-resistant strains [156]. A recent review by Almeida-Santos et al. (2021) discusses the advantages and challenges of using enterococcal derived bacteriocins against VRE and the diversity of bacteriocins produced by enterococci [157]. The review is an excellent collation of origin and classicisation of the enterocins highlighting that bacteriocins of enterococcal origin can be a tool against clinically relevant isolates but can also be exploited by clinically relevant enterococci during pathogenesis. Also of note is the number of enterocins localised to plasmids, 23 distinct bacteriocins [157]. A key tool in the implementation of bacteriocins as a therapy will be WGS, to identify novel bacteriocins with anti-VRE activity, and select peptides that do not have resistance mechanisms in clinical clades of enterococci.

A preliminary study of two bacteriocin-producing LAB, *Lactococcus lactis* MM19 (producing nisin Z) and *Pediococcus acidilactici* MM33 (producing pediocin PA-1), isolated from human feces, were found to be capable of reducing VREfm in a C57BL/6 mouse model by 1–2 logs [158]. Strains from non-pathogenic *Bacillus* species have attracted growing attention due to their safety and adaptability to various growing conditions [159]. The 1.9 kDa bacteriocin pumicilin isolated from *Bacillus pumilis* WAPB4, an environmental isolate, was active against both VREfs and MRSA and is heat (121 °C 15 min with 100% activity) and pH stable (pH 3.0 with 60% activity) supporting applications in the veterinary setting [160]. The effect was dose-dependent: at lower concentrations (20 AU/mL) the effect was bacteriostatic, while at a higher level (80 AU/mL), the effect was bactericidal. *Bacillus tequilensis* K1R was isolated from fermented dairy product kimchi, and was shown to produce a 4.6 kDa antimicrobial peptide active against VRE [159]. The 45-residue peptide is stable in a range of temperatures from 30–60ºC and pH 6.5–9 and the purified peptide displayed more potent activity than bacitracin and vancomycin of 16–32 μg/ml compared to > 128 μg/ml (MIC value).

Bacteriocin expression in commensal bacteria can influence niche occupation in the GIT, and bacteriocins delivered by commensals that occupy a precise intestinal niche may represent an effective therapeutic approach to specifically eliminate intestinal colonisation of VRE without disruption of indigenous microbiota [44, 145, 161]. An *E. faecalis* strain harbouring the sex–pheromone responsive plasmid pPD1 that encodes bacteriocin 21 was able to replace native enterococci in mice by elimination of bacteriocin-sensitive enterococci and non-pPD1 carrying strains of *E. faecalis* without disruption of the native microbiota [161]. When a derivative strain lacking the conjugative plasmid but carrying the bacteriocin gene was used in mice colonised with rifampicin resistant *E. faecium* V583, the bacteriocin-producing strain was able to successfully eliminate V583, suggesting that the introduction of a conjugation defective bac-21 producing strain can abolish colonisation by multidrug-resistant *E. faecalis* [161]. A recent study reported that a lanthipeptide produced by the gut commensal *Blautia producta* prevents VRE colonisation of the GIT in vivo with lower collateral damage than nisin-A, a similarly classed peptide [145]. The same study showed enrichment of lantibiotic gene expression in patients with reduced *E. faecium* suggesting a protective effect of lantibiotics on the human microbiota against VRE.

**Fig. 5:**
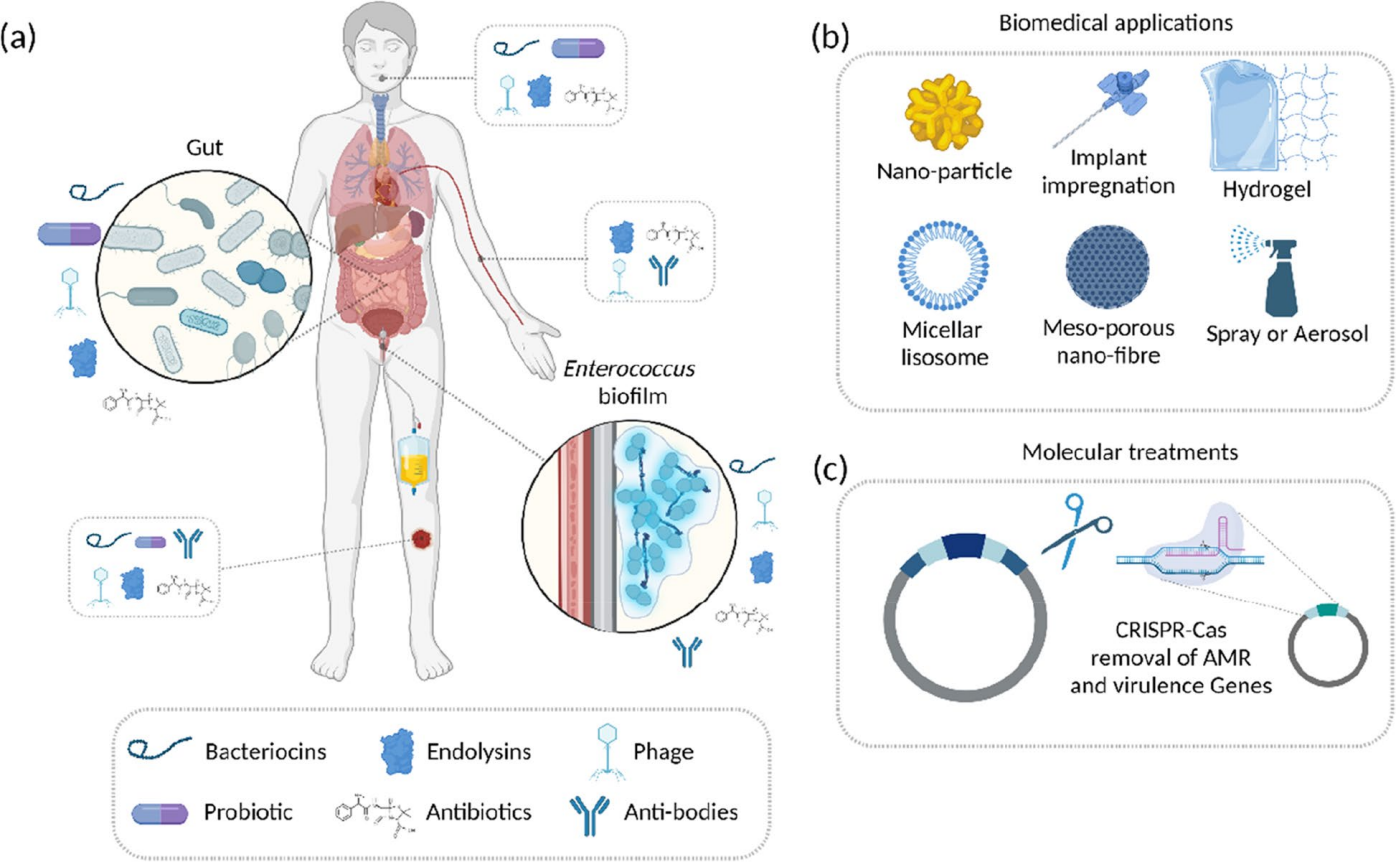
Summary of therapeutic options to combat VRE in the clinical setting. **a** Potential types of therapeutic interventions based on the stage and location of VRE infection. Bacteriocins can be applied topically and encapsulated to reach the gut [149, 156, 169–171]. Phage therapy can be used, with a phage cocktail being preferential, in combination with continued bacterial monitoring [172–175]. Phage derived lytic proteins are a therapeutic option against VRE for oral, wound, gut and bloodstream infections [176, 177]. Evidence for the effectiveness of probiotics are found in references 198–194 [178–184]. **b** Biomedical applications of anti-VRE molecules, which can increase the specificity and half-life and broaden the application range. Advances in therapeutic delivery strategies can overcome limitations of stability and specificity through controlled release of effecter molecules and impregnation of medical devices or fibrous dressings for wound care [168, 176, 185, 186]. **c** Novel molecular mechanisms to remove AMR genes from the microbiota are being investigated, and a promising avenue is using CRISPR-Cas systems to target critical AMR genes in mixed microbial communities [187]

Although in vitro studies suggest that bacteriocins against VRE are promising and evidence exists of their anti-VRE role within the microbiota, a number of limitations need to be overcome for the implementation of bacteriocins in disease treatment (Fig. 5) [44, 145, 156, 158–161]. Bacteriocins can be highly selective in nature, meaning bacteria often have innate resistance i.e. lack of bacteriocin binding ligand. Other innate systems are encoded among gram-positive bacteria, which can be upregulated in response to bacteriocins, these include altering the cell surface charge to deter bacteriocins, upregulation of two-component systems encoding ABC-transporters and sequestering proteins, as well as alternative expression of sigma factors, ultimately changing membrane stability and reducing bacteriocin affinity for the cell surface [162]. It is also possible that bacteria can harbour orphan immunity genes, which sequester bacteriocins, and proteases which inactivate bacteriocins genes suggesting a tailored approach may be needed for clinical efficacy which can be guided by WGS. Systemic use of bacteriocins could lead to the development of resistance via spontaneous non-synonymous mutations and varied frequencies have been documented, ranging from < 10^–9^ to 10^–2^ [162]. Interestingly, nisin resistant mutants of *S. aureus* displayed a 32-fold increase in susceptibility to gentamicin suggesting a combinatory regimen may be effective in returning efficacy to antibiotics [163].

The route of administration is another essential consideration, as bigger peptides face difficulties in absorption and the peptide nature of bacteriocins makes them sensitive to gut proteases. However, the gene-encoded nature of bacteriocins makes these molecules ideal for peptide engineering to improve their antimicrobial activity and physiochemical properties including their susceptibility to gut enzymes and target species [164, 165]. Thus, in vivo systems should focus on improving the stability and efficacy of these proteins in real environments [166]. Potential biomedical applications to overcome limitations of bioavailability of bacteriocins are shown in Fig. 5. Vaginally administered nisin was detected in blood after 1 h post-treatment, but levels declined within 12 h, suggesting high removal rates [167]. Comprehensive reviews of toxicity and regulation of bacteriocins and methods of targeted delivery exist but are beyond the scope of this review [149, 168].

##### Phage therapy

Bacteriophages are self-replicating entities which infect and can kill bacteria. Phage therapy was first described 100 years ago. Although it is still practised in countries including Georgia, Poland and Russia, it lost favour in Westernised countries with the advent of antibiotics [188]. Phages are highly specific for bacteria and thus safe for human use with minimal risk of secondary infection [189]. They cause negligible off-target changes in the host microbiota, are easy to isolate and can be effective against biofilms [190]. New phages against clinically relevant VRE have recently been isolated [174, 191, 192], but not many phages have been characterised with specific activity against VREfm. The bacteriophage IME-EFm5 was isolated from hospital sewage and is a rare example of an *E. faecium* lytic phage [177].

In terms of phage treatment of VRE infections, several studies have confirmed their potential. One of the first studies was conducted on a minimum lethal dose VRE bacteraemia mouse model, inoculated with 3 × 10^9^ colony forming 9 units (CFU) of *E. faecium* CRMEN44 [193]. A single intraperitoneal injection of phage ENB6, previously isolated from raw sewage, containing 9 × 10^9^ plaque forming units (PFU) rescued all VREfm-infected mice, even in the case of delayed treatment. Heat-treated phages did not help the survival of infected mice; hence it was concluded that the mechanism of action was phage function and not a consequence of a nonspecific immune response [193]. Similarly, another murine bacteraemia model study used a lytic phage, EF-P29, with broad host range against *E. faecalis* strains. A single intraperitoneal injection of 4 × 10^6^ PFU 1 h after VREfs infection rescued mice, and VREfs bacterial counts were 4 logs lower after 24 h in treated mice compared to the control group. The phage-treated group also saw a restoration of the microbiota towards the non-challenged control [194]. Compared with other studies, the dose of EF-P29 was much lower but could protect all mice in the treatment group. In another study, 1 × 10^10^ PFU of phage ΦVPE25 orally gavaged gave a three-fold reduction of *E. faecalis* in a gnotobiotic mouse model 24 h after administration [195]. A similar effect was confirmed when a high dose (10^9^ PFU) of phage EF-P29 was used [194]. A rebound of *E. faecalis* was observed after 24 h due to mutational resistance to ΦVPE25 with 80% of isolated clones resistant at 24 h, reaching 100% after day 9 [195]. Overcoming this hurdle involves the use of phage cocktails. Administration of a two-phage cocktail rescued septic mice with multiple organ invasive infection with a 5-log reduction, and phage administration to healthy controls did not significantly alter the murine microbiome alpha and beta diversities. A phage and ampicillin combination led to the most significant decrease in bacterial titre in distal and proximal tissues of the mice and rescued TNF-alpha levels towards healthy controls [196].

Enterococci are known to form biofilms, which prevent antimicrobial compounds from penetrating the cells and eradicating the bacteria. However, phages can target bacteria within biofilms [197]. The phage EFDG1 was isolated from sewage and proven to be active against *E. faecalis* V583 biofilms in in vitro and ex vivo experiments. Additionally, EFDG1 could infect both *E. faecalis* and *E. faecium* strains tested in the study [198]. In a subsequent publication, the authors described resistance to EFDG1 and a second phage, EFLK1, was isolated, that could eradicate EFDG1 resistant mutants [173]. Both phages shared 60 core genes, approximately 28% shared genomically, but EFDG1 killed bacteria in the logarithmic stage of growth and phage EFLK1 was more effective when cells were in the stationary phase. The authors suggest that phages differ in affinity or have different receptors altogether. Notably, a two-phage cocktail proved efficient in killing both parent V583 and EFDG1-resistant mutants separately, as well as in mixed culture, and it also proved effective at killing the biofilm of V583 [173]. When these two phages were combined with ampicillin, additional benefits were observed, such as the highest decrease in the number of bacteria in organs, including the liver and heart, in VREfs V583 infected mice, and the phage cocktail alone was capable of completely reversing a 100% mortality trend in the infected mice [199]. A separate study identified phage-resistant mutants containing SNPs in cell wall synthesis proteins resulting in reduced phage absorption, but the mutants had enhanced susceptibility to ampicillin (2–5 fold reduction in MIC) and, notably, daptomycin, highlighting the potential for combination approaches [200]. The authors also describe synergy between phage and daptomycin against *E. faecium.* Recently, a personalised phage cocktail was clinically used to cure a 1-year-old child of recurrent invasive abdominal *E. faecium* infection post liver transplant [201]. 5.2 × 10^8^ PFU/mL was intravenously administered at 2 mL/kg body weight (BW) twice daily – C-related protein levels dropped within 24 h along with improved clinical status, and routine swab screening could not detect patient VREfm for the rest of the hospital duration.

Unlike antibiotic resistance, phage therapy offers several solutions for the treatment of emerging phage-resistant strains, including the isolation of alternative phages which is a relatively easy and straightforward process, the use of phage cocktails, and phage training which enables the improvement of existing phages in terms of infectivity and host range as discussed by Hill et al. (2018). The prospect of phage engineering is discussed by Mahler et al. (2023), and has been applied to engineer a lytic phage from a lysogenic background to eliminate *E. faecalis* [202, 203]. WGS can drive the development of “designer-phage” to eradicate MDR bacteria by identifying phage virulence determinants which can be subject to phage rebooting without the virion genes [204]. As touched on earlier, patient-specific pathogen sequencing can be expanded to phage therapy in a sequencing-based tailored phage therapy workflow moving phage therapy into the 21st century [205]. An in-depth review of the infrastructure and framework required to translate phage-therapy into a more readily available therapeutic option touches upon the effect genomics can have on this pathway [206]. These include having a database of sequenced phages with data on pathogens and phages, with in-depth functional characterisation of virulence, phage-host determinants (receptors), prediction of phage defence systems and algorithms using phage features mapped to pharmacokinetics and pharmacodynamics or immunogenicity to develop effective phage formulations based on sequencing data [206].

Phages have numerous roles within the microbiota other than infective elimination of hosts, such as HGT, hence recent research is investigating isolated phage-derived endolysins as a therapeutic alternative to the phage itself. A zinc dependant amidase endolysin, LysEFm5, has been described with activity against VREfm, although activity was not quantified at a range of pH inclusive of conditions similar to urine [177]. These enzymes are susceptible to genomic engineering, plug-and-play between cell-wall binding domains and activity domains, and a comprehensive review of their benefits and limitations has been previously published [176]. With the expansion of metagenomic datasets, it is possible to synthesise endolysin DNA for heterologous expression, for which 1000s have been discovered in uncultured phage genomes [207].

##### Commensals

**Fig. 6:**
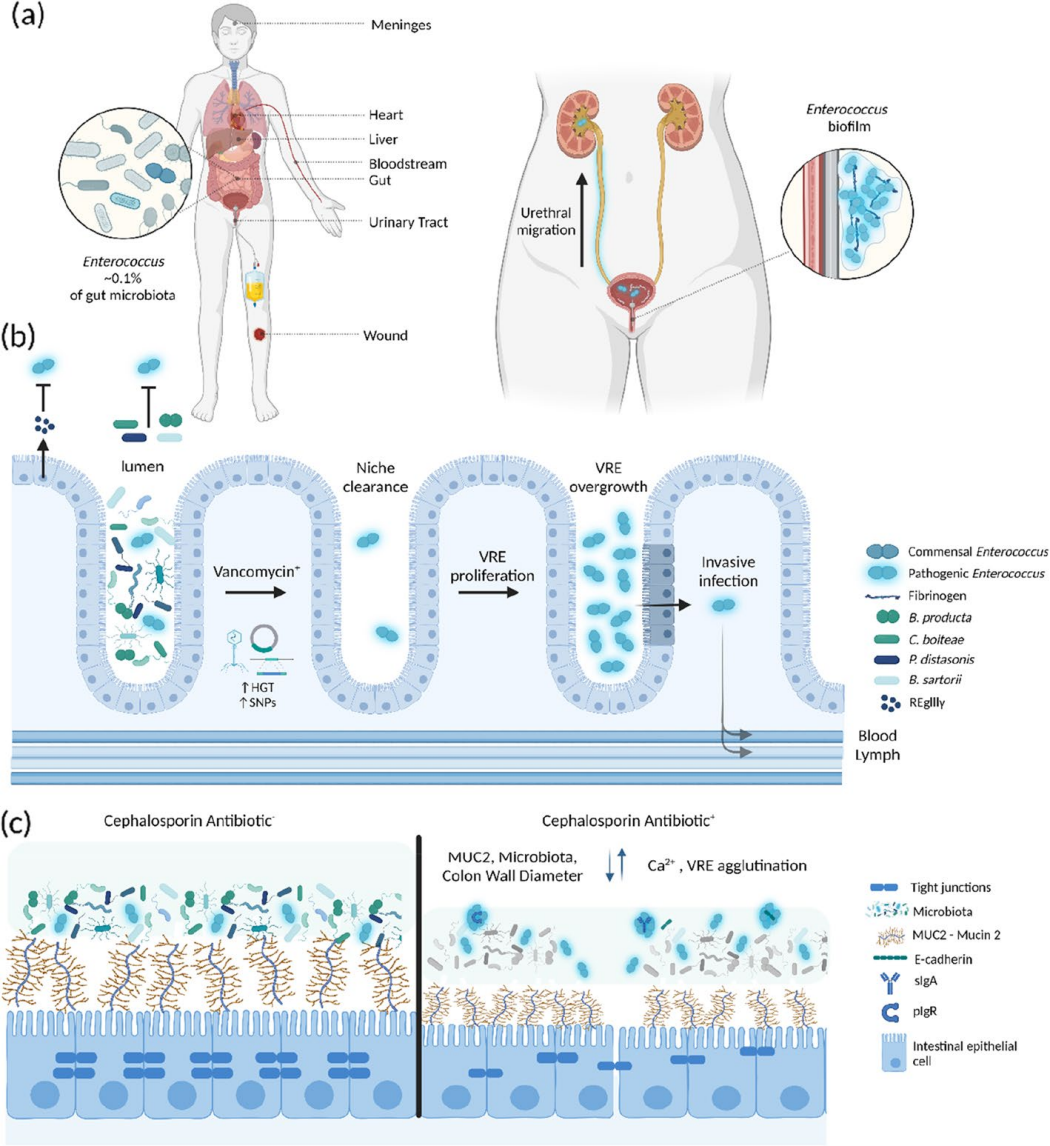
Aetiology of VRE invasive infection. **a** Enterococci typically represent ~ 0.1% of the human gut microbiota. Invasive infections can occur at various body sites, but one of the main routes in infection other than the GIT is through UTIs. Environmental *Enterococcus* spp. can migrate and form robust biofilms on urinary catheters incorporating fibrinogen into a matrix where persistence can result in invasive infection and urethral migration to infect the kidney. **b** Invasive infection can occur through a weakened GIT lining, where translocation can lead to systemic disease of the blood or lymph systems. The presence of commensals prevents VRE colonisation through multiple modes (1) production of REglllY by enterocytes stimulated by commensal gram-negative bacteria in the GIT [144]. (2) Direct inhibition by the production of bactericidal peptides, eg. lanthipeptide production by *Blautia producta* and pheromone production by commensal enterococci [44, 145]. (3) Preoccupation of shared niche. (4) Cooperating commensals allow a microbiota-wide reduced sensitivity to ampicillin, allowing peptide producing strains to confer colonisation resistance to VRE [146]. **c** The administration of cephalosporin antibiotics significantly reduce the microbiota and alter the host physiology decreasing the thickness of the colon wall, reducing the mucus layer and MUC2 (Mucin-2) and damaging the integrity of tight junctions via elevated Ca^2+^ [147]. Host factors such as E-cadherin, sIgA and pIgR also cause agglutination of *E. faecium* after an antibiotic induced “bloom” to protect the host and shed clustered VRE into the lumen [147]

Although VRE can be isolated from skin, urinary tract or oral cavity, most enterococci, including VRE, primarily colonise the GIT. These species present only a minor part of the gut microbiota in healthy individuals. Increasing enterococcal populations beyond sub-dominant threshold levels in the gut leads to infection [208, 209]. This occurs when antibiotics deplete the host microbiota, and MDR VRE flourishes in the vacated niche (Fig. 6). One such mechanism of microbiota protection involves the production of lipopolysaccharide and flagellin by gram-negative bacteria, including anaerobes, stimulating the production of REGIIIγ, a C-type lectin with antimicrobial activity against VRE. The introduction of antibiotics causes a decrease in the gram-negative fraction of the microbiota, followed by decreased production of REGIIIγ and the subsequent dominance of VRE in the GIT [210]. Additional factors contributing to the development of enterococcal infection include other severe diseases, including cancer and subsequent treatment where prophylactic antibiotic administration decreases bacterial counts but selects for enterococcal species or the potential risk for vascular catheter-associated infection due to long-term use, neutropenia, and urinary catheters [211–213]. Invasive VRE entry to the bloodstream occurs by translocation from the GIT to the blood or lymph system or through invasive medical procedures [214]. As mentioned, reducing VRE colonisation overgrowth and carriage has become necessary in reducing the risks of infection and invasion [22].

Colonisation resistance is a dynamic phenomenon whereby the native non-antibiotic treated commensal gut flora serves as a natural barrier against pathogen colonisation resulting from competition for nutrients, physical occupation of niches, production of antimicrobial compounds (e.g. bacteriocins), secondary metabolites, and modulation of the host immune response against pathogenic bacteria. However, this barrier becomes imbalanced due to antibiotic treatment, and as early as the mid-1950s, the loss of the majority of obligate anaerobes was attributed to susceptibility to infection. The application of vancomycin in mice caused a decrease in Bacteroidetes and the dominance of VRE in the small and large intestines even after the termination of antibiotic treatment [215]. In a murine model, the administration of vancomycin promoted VRE colonisation by disrupting the barrier formed by the intestinal microbiota and provided a selective advantage for resistant bacteria such as VRE to the detriment of the normal flora [183]. Intestinal domination by VRE precedes bloodstream infection [216]. Administration of antibiotics leads to selective survival of resistant species, and increases the susceptibility to further infection, caused by the removal of bacterial species that provide colonisation resistance. Indeed, the density of colonisation reached by VRE can lead to high transmission rates within a hospital setting [216]. Since colonisation and proliferation are critical steps in infection development and transmission, prevention of overgrowth and persistence provided by the gut microbiota is an attractive non-antibiotic alternative to minimise the colonisation and persistence of VRE [217]. As mentioned, the obligate anaerobe *B. producta* has lantibiotic-mediated anti-VRE effects on the microbiota [145]. Interestingly, the presence of *Blautia* spp. also correlated with reduced enterococcal abundance in a case–control study of hospitalized VRE patients compared to control [218].

Faecal microbiota transplantation (FMT) represents the transfer of gut microbiota from a healthy donor to a compromised recipient to remedy the imbalance in the microbial community, a characteristic of several gut disorders. Although variable success rates are reported in the literature, this approach is promising, albeit with some concerns, such as protocol, donor evaluation and standardisation [219]. FMT for the treatment of *C. difficile* and ulcerative colitis exists, but there is also evidence of the efficiency of this treatment for the prevention and clearance of VRE [220]. The introduction of a donor microbiota to VRE colonised mice showed that obligate anaerobes from the genus *Barnesiella* were significantly correlated with the elimination of the pathogen, suggesting that recovery of critical components of the microbiota is a crucial factor for successful VRE elimination. After 15 days, VRE CFUs/10 mg of faeces were below the detection limit compared to the phosphate buffered saline (PBS) control of 7–8 log CFUs/10 mg [221]. Also, transplant patients who did not develop VRE contained higher levels of *Barnesiella,* suggesting a protective effect [221]. FMT was also influential in eliminating concurrent *Klebsiella pneumoniae* and VRE infection in mice, where VRE was cleared in 60% of mice and reduced by 3 logs in the remaining 40% of infected animals [222]. It is possible to predict the recurrence of *C. difficile* infection based on genus-level relative abundances of operational taxonomic units (OTUs) using 16s rRNA sequencing, a method that could be used to reduce the percentage of non-responder FMT recipients for VRE [223]. This suggests WGS may be critical in matching donor to recipient to improve clinical outcome by reducing exclusion effects on incoming strains from the host microbiota [224].

The presence of specific species and their cooperation in the microbiota is essential for maintaining colonisation resistance [146]. The authors firstly determined a mix of 7 strains that could prevent VRE colonisation in ampicillin-treated mice (*Blautia spp., B. producta, Clostridium bolteae, Eubacterium dolichum, Akkermansia muciniphila, Bacteroides sartorii* and *Parabacteroides distasonis*). Loss of colonisation resistance occurred in mice treated with bacterial mixtures deficient in either *C. bolteae* or *B. producta*, suggesting their role was essential in establishing colonisation resistance. The whole consortium was needed to re-establish colonisation resistance in ampicillin-treated mice: *B. sarotrii* and *P. distasonis* are highly ampicillin-resistant and they inactivate ampicillin enabling successful engraftment of *C. bolteae*, which subsequently supports the colonisation of *B. producta* [146].

*E. faecalis* V583 showed limited growth ability in a consortium of GI flora that included commensal enterococci. This incompatibility was traced to a pheromone produced by commensal enterococci, cOB1, which is efficient in killing VREfs V583. The pheromone cOB1, a resulting product of lipoprotein EF2496’s cleaved leader sequence, is pumped extracellularly by plasmid free commensal enterococci. This 8-residue peptide pheromone increases transcription of the plasmid pTEF2 in *E. faecalis* V583 100-fold, resulting in cell lysis. Cell lysis occurred through an unknown mechanism but was dependent on a plasmid-bound IS, for which spontaneously resistant mutants of V583 to cOB1 had lost the IS element. This suggests plasmid proteins acting *in trans* coupled with imprecise excision of IS resulted in cell lysis [44]. It has been hypothesised that induction of conjugative functions on pTEF2, induced by cOB1 leads to lethal cross-talk between mobile elements on the plasmid and IS elements on the chromosome, facilitating an imprecise excision and a double strand break in the chromosome, causing cell death [225]. The susceptibility of V583 to killing by commensal strains provides strong evidence that they rarely occur in the same niche and that V583 has proliferated in habitats in which commensal strains are excluded. This provides clear evidence supporting the hypothesis that MDR hospital strains and commensal enterococci cannot occupy the same habitat and reveals differences in the colonisation properties of hospital and commensal lineages. This brings new hope for therapies that can preserve the native enterococcal flora and serve as a barrier to colonisation by hospital-adapted lineages [225].

A recent study uncovered that fructose depletion restricts VRE colonisation using a commensal bacterial consortium [226]. Subsequent downstream analysis using RNA-seq identified an *Olsenella sp.* that could reproduce the effect of the consortium and protect from vancomycin induced susceptibility to VRE in a mouse model [226]. *Olsenella* treated mice saw a 2-log reduction in VRE from CFU counts of faeces [226]. Identifying anti-pathogenic microbes in a microbiota through 16s rRNA sequencing with a computational workflow is an emerging field of study but has yet to be applied at WGS resolution [227].

##### Probiotics

Probiotics are “live microorganisms which when administered in adequate amounts confer a health benefit on the host” [228]. The era of probiotic expansion began in the early 2000s, with a growing number of studies reporting health benefits from the intake of live microorganisms in the prevention or therapy of various conditions. The most widely explored application of probiotics is in treating gut malfunctions. However, the efficacy of probiotics for other conditions, such as allergy, obesity, and diabetes, exists [229]. It has been shown that probiotic administration can enhance resistance to pathogenic bacteria [230]. Since the gut represents a VRE reservoir, from where translocation can occur, the application of probiotics finds its validity in controlling VRE proliferation in the GIT and its subsequent dissemination throughout the body. Enhancing intestinal resistance against VRE by probiotics could be a prophylactic means of decreasing the risk of VRE invasive infection. In a murine model, 10^9^ CFU of the probiotic *L. paracasei* CNCM I-3689 significantly reduced VRE by up to 6 logs below the limit of detection, even though the strain does not directly inhibit VREfs V583 in vitro, instead it modulates the host intestinal response by increasing production of cathelicidin, an AMP, and decreasing levels of the pro-inflammatory cytokine IL-12 [217]. *Lacticaseibacillus rhamnosus* did not significantly reduce VRE counts compared to the control. Additionally, the administration of the *L. paracasei* strain improved microbiota recovery after clindamycin-induced dysbiosis, helping to maintain Bacteroidetes levels [217]. A study performed on mice showed that 10^7^ CFU of orally administered *Bacillus coagulans* strain for four days decreased the density of one VanB-resistance carrying VRE strain in stools, but not the second VanB or VanA resistance-carrying strain highlighting the strain-dependant nature of both effector and effected bacteria [231]. A cell-wall preparation of *E. faecalis* EC-12 decreased the numbers of inoculated VRE in chickens 3 days after the pathogen challenge [232]. This preparation also increased the production of total and VRE-specific IgA and IgG, respectively. Additionally, a selected undefined *Lactobacillus* spp. showed a protective effect based on a potential competitive exclusion in the gut of chickens [232]. A similar study showed that the administration of *E. coli* Nissle to VREfm-colonized mice failed to decrease VREfm density in the gut. In contrast, administration of *L. rhamnosus* Lcr35 lowered VREfm density, albeit not to a level of significance. Additionally, 5 weeks of oral intake of 10^9^ CFU Lcr35 in 9 VRE patients had no significant effect on VRE levels [183].

A limited number of studies have investigated the efficacy of probiotic strains in combating VRE colonisation in the gut. Some positive effects of probiotic administration in VRE colonised humans and animals have been documented, but these effects remain strain-specific [184]. A double-blind, randomised, placebo-controlled study found *L. rhamnosus* GG (LGG) increased VRE clearance among all patients from the stool after four weeks, while in the control group who received standard pasteurised yoghurt, only one out of 12 subjects cleared VRE. Patients in this group who subsequently received yoghurt with the probiotic strain shed the VRE [181]. LGG, was assessed for its ability to eradicate VRE in a randomised clinical study among colonised children and a temporary significant reduction was observed after 3 weeks of 3 × 10^9^ CFU consumption daily, where 20/32 carriers lost VRE (*P* = 0.002), which was assessed by rectal swabbing [233].

A more recent study assessed the same probiotic culture in preventing VRE colonisation in adults with comorbidities [234]. Up to 2–5 × 10^10^ CFU/g LGG was administrated in a gelatine capsule for 14 days, and stool samples were analysed for 56 days. In contrast to the previous study, this one reported no difference in VRE colony counts between the control and treatment groups, although the probiotic strain exhibited bactericidal effects in in vitro studies performed against four strains of VRE. Also, no decline in VRE colony counts were observed over time in subjects who received LGG. Several factors could have contributed to these contrasting results, including the shorter administration period in the case of the capsule, the presence of comorbidities, different formulations of the LGG preparation, and administration of antibiotics in the treatment group. Concomitant administration of antibiotics within the trial group resulted in the inability to recover viable LGG, although DNA was detected [234].

Another study in 2010 found similar results where a multispecies probiotic powder, consisting of 10 distinct species, administered twice daily failed to prevent colonisation of ampicillin-resistant enterococci [235]. Although there are some limitations to this study, including the administration of antibiotics that persist in the body may have reduced the efficacy. There was also a low frequency of probiotic intake, with patients taking the probiotic 58% of days of duration of stay [235]. A 2016 meta-analysis of probiotic and synbiotic therapy encompassing 30 trials, identified a reduction in infectious complications but the authors acknowledge that more large-scale and adequately powered clinical trials are required to confirm observations made from the study [236]. Exploiting probiotic bacteria beyond use for the patient is possible, where a 2023 cluster-randomized crossover study found routine surface cleaning in 18 non-ICU wards with a probiotic, consisting of multiple *Bacillus* species, was similar in efficacy to soap-based and disinfectant-based cleaning strategies in preventing HAI [108].

Utilising metagenomics for the advancement of probiotics was discussed in a review by Suez et al*.* (2020). Beyond measuring the effects on the microbiota through functional and compositional genomics, strain-level functional genomics could be a tool to predict probiotic engraftment resulting in patient tailored probiotic regimens [237, 238]. Engraftment of *Bifidobacterium longum* AH1206 was studied by Maldonado et al*.* (2016) and they found the microbiota was deterministic for strain persistence in the gut [239]. An underrepresentation of carbohydrate utilising genes in the microbiota of participants favoured colonisation, whereas establishment was prevented through competitive exclusion by phylogenetically related resident microbes, suggesting colonisation was determined by niche and resource availability [239]. Interestingly, these findings align with competitive exclusion of VRE mentioned previously [226]. These findings suggest WGS could be a tool for predicting engraftment of probiotics based on the carrier state of the subject's microbiota and if a functional niche is vacant.

A recent tool has also been described to predict probiotic properties in WGS data for the rapid identification of putative probiotic candidates [240].

##### Vaccine

A detailed account by Micoli et al. (2021) for the role vaccinology could play in combating AMR, but also the limitations, has recently been published [241]. As mentioned previously, there is a distinct genomic fingerprint between CL and NC clades of *E. faecium* but admixture between the clades arises.

The advancement of new technologies in reverse vaccinology (RV), structural vaccinology and the design of outer membrane vesicles as state-of-the-art vaccine technology are emerging. RV is driven by genomics, to find novel antigenic proteins to specifically target *E. faecium*. Bioinformatic algorithms such as New Enhanced Reverse Vaccinology Environment (NERVE) are accelerating RV [242, 243]. The ability to predict cellular localisation of proteins, solubility, immunogenicity and compute homology to known antigens can be done on a desktop [243]. A pan-genome approach has previously been used by Maoine et al. to develop the concept of a group B *Streptococcus* vaccine, where 3 of the 4 tested antigenic proteins were part of the accessory genome [244, 245]. This is a plausible method for tackling enterococcal clinical transmission and infection due to clade specific markers among clinical isolates. Kalfopoulou and Huebner (2020) describe the recent advances of enterococcal vaccine developments and highlight the capsular polysaccharide and protein targets amongst the virulent strains. These proteinaceous vaccine candidates include virulence associated traits such as *GelE*, *PBP5*, *SagA* and *Ace* [246–250]. In an infective endocarditis rat model, an anti-*Ace* immunotherapy against *E. faecalis* showed a significant reduction in Log_10_ CFU/gm (3.8 ± 1.4) and resulted in a 20% overall invasive endocarditis compared to 83% for the control group in a passive immunisation experiment [246]. A similar result was observed for active immunisation against the virulence factor, where 65% of the test group did not develop infective endocarditis and all immunized mice had high anti-Ace antibody titers (1: > 50,000) [246]. LysM, a peptidoglycan-binding protein found on 90% of plasmids among clinical isolates represents a targeted, plasmid specific antigenic protein for which anti-LysM antibodies raised against recombinant LysM resulted in a significant reduction of CFU/mg in a bacteraemia mouse model [9, 247]. The genomic plasticity of enterococci may lead to complications on the road for an effective vaccine against MDR VRE – which could be further complicated by the selective pressure of the immune system. In this instance, a vaccine that encompasses multiple clade specific antigens to reduce the likelihood of escape mutants could suffice [245].

##### Repurposing of drugs

Repurposed non-antimicrobial drugs were tested to show their ability to prevent GIT colonisation by VRE. The ability to produce biofilms is one of the key factors enabling efficient VRE colonisation. Ebselen, a drug usually used in the treatment of a range of diseases, such as cancer, stroke, atherosclerosis, neuropathy and bipolar disorders, showed activity in decreasing biofilm formation and contributed to the in vivo reduction of VRE in the cecum and ileum of mice [251]. The same molecule was shown to have anti-*C. difficile* activity by disrupting redox homeostasis, preventing recurrent infection and promoted microbiome recovery after antibiotic treatment in mice [252, 253]. The same research group showed that the anti-inflammatory rheumatoid arthritis drug auranofin portrayed remarkable activity against VRE, most effective at low doses, with no detectable resistance developed and prevented VRE colonisation in an in vivo challenge study [251, 254]. Similarly, the group showed that at clinically achievable doses, the drug can prevent *C. difficile* recurrence but was ineffective at higher doses. The authors suggest this may be due to off-target effects of auranofin on the microbiota or the drug’s anti-inflammatory properties highlighting the complexity associated with repurposing drugs as antibiotic alternatives [255, 256]. These novel approaches present a radically different direction for treatment of GIT colonisation with VRE, and could become financially viable options to prevent spreading and complications of VRE if used correctly.

##### Artificial intelligence

Advances towards the application of artificial intelligence in drug development and discovery have been made since the turn of the decade and are hard to ignore. These tools include the accurate prediction of protein structure from a protein sequence, generative AI models producing never-before seen protein sequences with structural homology to functional proteins, and the discovery of antimicrobial peptides by mining the 3D space of proteins [257–263]. The first evidence of a machine learning guided therapeutic peptide *in* silico screen identified peptides with activity against VRE in 2008 [264]. In this study, a neural network trained on existing MIC values and protein structures predicted antimicrobial activity. A validated model was used to screen 100,000 putative cationic antimicrobial peptides. MIC values were determined against multiple VRE strains with varying values for *E. faecali*s (3.1 – 241 μM) and *E. faecium* (1 – 226 μM). Strikingly, 94% of the top 50 candidates had antimicrobial activity [264]. A more in-depth study mining the 3D space of proteins identified hexameric peptides with efficacy against MDR pathogens, comparable to penicillin, with minimal toxicity, and low frequency of resistance [262]. Although VRE were not tested, the peptides were able to reduce *S. aureus* by 3 logs in an acute pneumoniae model in mice, which shows a positive direction for AI guided therapeutic interventions against gram positive infections [262].

#### Concluding remarks

As reported by the World Health Organisation, vancomycin-resistant enterococci, are recognised as high-priority pathogens for which new efficient therapeutics are urgently needed. As pathogens, VRE represent MDR strains which have a fascinating ability to withstand environmental stressors, adapt rapidly and disseminate globally. To better understand the occurrence, distribution and dissemination of vancomycin resistance, WGS and comparison of genomic information has been successfully employed. It has also facilitated an understanding of the routes of pathogen transfer among patients, healthcare workers and non-hospital environments, as well as the role of the mobilome among VRE. Additionally, genomic information has provided insight into differences in genomic content between hospital and communal strains and the presence of specific genetic determinants that confer the ability of strains to survive in these very different environments successfully. On the other hand, the prevention of GIT colonisation and inhibition of VRE proliferation in the gut is a crucial step in controlling its transfer to the bloodstream and the subsequent development of VRE infections. For this reason, a promising approach is to enhance the gut microbial shield through the use of selected strains of probiotics or consortia of commensal microorganisms with proven inhibitory activities against VRE colonisation. In treating systemic infections, combinatorial treatments involving conventional antibiotics may result in synergistic antimicrobial effects, but studies investigating antibiotic alternatives have also shown promise. In particular, bactericidal molecules, such as bacteriocins, phage therapy or their endolysins, provide hope in overcoming the decreasing efficacies of antibiotics. Future studies should focus on developing these therapies by employing a tailored approach for use in clinical settings to circumvent future HGT events among clinically relevant isolates and the ever-expanding pool of AMR genes.

## Data Availability

Scripts used to align sequences and construct the phylogenetic tree are stored in https://github.com/DEHourigan/Efaecium_review_2023.

## Abbreviations

ANI: Average nucleotide identity
CA: Community associated
CC: Clonal complex
CFU: Colony forming units
cgMLST: Core genome multi-locus sequence type
CNS: Central nervous system
CRISPR: Clustered regularly interspaced short palindromic repeats
DNA: Deoxyribonucleic acid
DRE: Daptomycin resistant Enterococci
FDA: Food and drug administration
FMT: Faecal microbiota transplantation
GI: Genomic island
GIT: Gastrointestinal tract
HA: Hospital associated
HAI: Hospital associated infection
HGT: Horizontal gene transfer
IS: Insertion sequence
LAB: Lactic acid bacteria
LGG: *Lacticaseibacillus rhamnosus* GG
MDR: Multi-drug resistant
MGE: Mobile genetic element
MIC: Minimum inhibitory concentration
MLST: Multi locus sequence type
MRSA: Methicillin resistant *Staphylococcus aureus*
NSG: Niche specific genes
PFGE: Pulsed field gel electrophoresis
PTS: Phosphotransferase systems
RV: Reverse vaccinology
SCV: Small colony variant
ST: Sequence type
UTI: Urinary tract infection
VF: Virulence factor
VRCD: Vancomycin resistant *Clostridium difficile*
VRE: Vancomycin resistant Enterococci
VREfm: Vancomycin-resistant *Enterococcus faecium*
VREfs: Vancomycin-resistant *Enterococcus faecalis*
VRSA: Vancomycin resistant *Staphylococcus aureus*
VSEfm: Vancomycin susceptible *Enterococcus faecium*
VSEfs: Vancomycin susceptible *Enterococcus faecalis*
WGS: Whole-genome sequencing
